# *Lactobacillus rhamnosus* GG ameliorates osteoporosis in ovariectomized rats by regulating the Th17/Treg balance and gut microbiota structure

**DOI:** 10.1080/19490976.2023.2190304

**Published:** 2023-03-20

**Authors:** Mengyu Guo, Huanjin Liu, Yinting Yu, Xingyu Zhu, Hui Xie, Chenxu Wei, Chunmei Mei, Yun Shi, Nong Zhou, Kunming Qin, Weidong Li

**Affiliations:** a School of Pharmacy, Nanjing University of Chinese Medicine, Nanjing, China; b Jiangsu Key Laboratory of Chinese Medicine Processing, Engineering Center of State Ministry of Education for Standardization of Chinese Medicine Processing, Nanjing University of Chinese Medicine, Nanjing, China; c School of Pharmacy, Jiangsu Ocean University, Lianyungang, China

**Keywords:** Osteoporosis, OVX, *Lactobacillus rhamnosus*, Th17/Treg, gut microbiota, gut barrier

## Abstract

**Background:**

With increasing knowledge about the gut – bone axis, more studies for treatments based on the regulation of postmenopausal osteoporosis by gut microbes are being conducted. Based on our previous work, this study was conducted to further investigate the therapeutic effects of *Lactobacillus rhamnosus* GG (LGG) on ovariectomized (OVX) model rats and the immunological and microecological mechanisms involved.

**Results:**

We found a protective effect of LGG treatment in OVX rats through changes in bone microarchitecture, bone biomechanics, and CTX-I, PINP, Ca, and RANKL expression levels. LGG was more advantageous in promoting osteogenesis, which may be responsible for the alleviation of osteoporosis. Th17 cells were imbalanced with Treg cells in mediastinal lymph nodes and bone marrow, with RORγt and FOXP3 expression following a similar trend. TNF-α and IL-17 expression in colon and bone marrow increased, while TGF-β and IL-10 expression decreased; however, LGG treatment modulated these changes and improved the Th17/Treg balance significantly. Regarding the intestinal barrier, we found that LGG treatment ameliorated estrogen deficiency-induced inflammation and mucosal damage and increased the expression of GLP-2 R and tight junction proteins. Importantly, 16S rRNA sequencing showed a significant increase in the Firmicutes/Bacteroidetes ratio during estrogen deficiency. Dominant intestinal flora showed significant differences in composition; LGG treatment regulated the various genera that were imbalanced in OVX, along with modifying those that did not change significantly in other groups with respect to the intestinal barrier, inflammation development, and bile acid metabolism.

**Conclusions:**

Overall, LGG ameliorated estrogen deficiency-induced osteoporosis by regulating the gut microbiome and intestinal barrier and stimulating Th17/Treg balance in gut and bone.

## GRAPHICAL ABSTRACT

**Figure:**
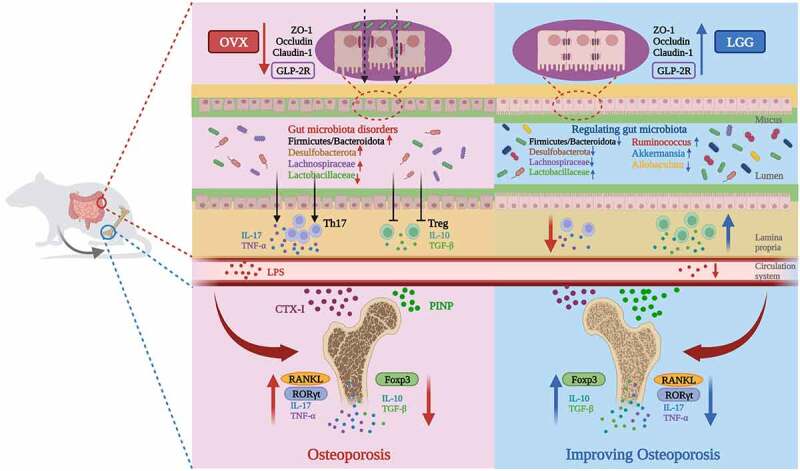
**Graphical summary.** LGG alleviates osteoporosis in ovariectomized rats by modulating the gut microbiome and intestinal barrier and improving Th17/Treg balance in gut- bone.

## Introduction

With the aging population, the frequency of osteoporosis is increasing, which usually decreases bone strength, mass, and density and increases fragility, often leading to fractures. Although the incidence of fractures varies greatly from country to country, women over 50 years of age have an average risk of up to 50%.^1^ However, osteoporosis is often effectively untreated owing to the cost and side effects of approved drugs, which underscores the urgent need to develop inexpensive and safe interventions.^2,3^

The gut microbiome has been shown to have a profound effect on bone quantity, quality, and overall strength in many clinical studies.^4–6^ The gastrointestinal tract contains the highest concentration of immune cells that communicate with the microbial community, triggering the release of metabolites or immune responses, which affect the immune system directly.^7,8^ The intestinal epithelial barrier plays a crucial role in separating the internal structures from harmful antigens and pathogens and performing immune protective functions.^9^ Since intestinal epithelial cells are sealed by tight junction proteins (TJs),^10^ such as ZO-1, claudin-1, and occludin, the intestinal microbiota can alter the expression and distribution of TJs, thus altering the permeability of the intestinal barrier.^11^ Impairment of the intestinal barrier allows microorganisms to enter the subepithelial structures from the intestinal metastatic lumen, and trigger an inflammatory response. In osteoimmunological studies, the balance of Th17/Treg and related inflammatory factors have been shown to be closely related to bone metabolism dysregulation.^12^ Sex steroid deficiency leading to intestinal barrier dysfunction and subsequent increase in circulating lipopolysaccharides (LPS) and CD4+ T cells has been suggested as an important mechanism for the development of osteoporosis in postmenopausal women.^13,14^ One mechanism of probiotic function is to increase the strength of the intestinal epithelium by upregulating TJs, reducing antigen presentation and activation of intestinal immune cells,^13,15^ which can lead to changes in the bone mineral density.^16–18^

The immunomodulatory potential of LGG in bone health through the systemic Th17/Treg immune regulation has been reported.^19^ However, the mechanism by which LGG ameliorates osteoporosis through Th17/Treg has not been well studied. Our previous study found that the occurrence of osteoporosis in the OVX rat model was accompanied by changes in the intestinal microecology, especially the reduction of Lactobacillus is closely associated with osteoporosis, and elevated expression of serum TNF-α, IL-6, and IL-17.^20^ To further investigate any correlation between the pro-inflammatory factor changes in OVX and Th17/Treg and the mechanism of probiotic LGG treatment, we focused on the alteration of the gut microbiome and changes in the gut barrier and associated metabolites by administration of LGG. From this perspective, it showed the immune potential of LGG to ameliorate osteoporosis through Th17/Treg immune regulation in ovariectomized rats. It was concluded that estrogen deficiency impaired the intestinal barrier, and increased intestinal permeability and Th17/Treg imbalance in OVX rats. This resulted in increased expression of pro-inflammatory factors in the intestine and bone, disrupting bone metabolism. LGG treatment adjust the gut microbiome structure and metabolism, improve the inflammatory status, and ameliorate osteoporosis.

## Results

### LGG (ATCC7469) characteristics

**Figure 1.**
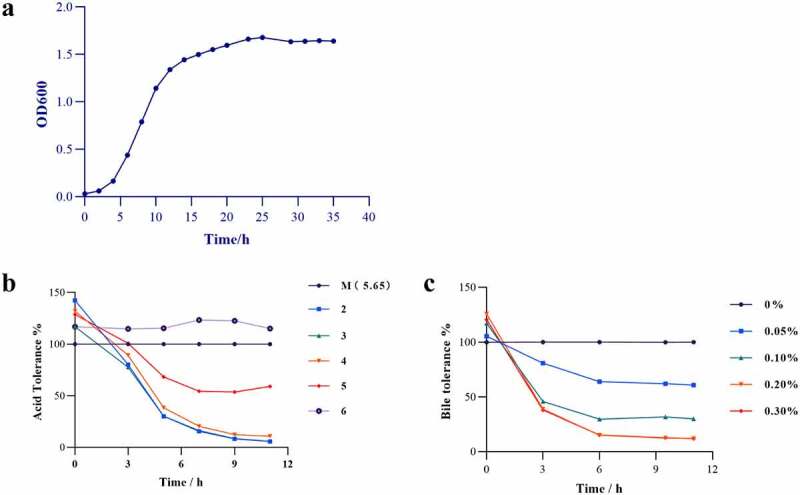
*Lactobacillus rhamnosus* GG (LGG) (ATCC7469) characteristics. (a) LGG growth curve. (b) Survival rate of LGG in medium with different pH. (c) Survival rate of LGG in different concentrations of bile salts medium.

The ability to adhere to and colonize the gut, an essential characteristic of probiotic microorganisms to be effective in the host, is closely related to the species and strain. LGG requires epithelial cell mediation to function,^21^ and its adhesion properties and colonization ability have already been studied.^22^ Here we examined the basic properties of ATCC7469, a wild-type strain of LGG. LGG was in the logarithmic phase from 2–12 h (Figure 1(a))when the bacteria grew exponentially in a steady geometric progression curve, and the bacterial morphology and biological activity were more typical and sensitive to external environmental factors. Follow-up experiments and drug delivery were performed using active bacterial organisms during the stabilization phase.

Gastric acid, bile salts, and various digestive enzymes in the gastrointestinal tract have potential inactivating effects on probiotics, and resistance to these external factors to ensure a viable number of bacteria is a prerequisite for probiotics to colonize and function. Growth of LGG was inhibited to different degrees under acidic conditions and concentrations of bile salts (Figure 1(b,c)). The number of viable bacteria in the artificial gastric fluid significantly decreased, with a 29% survival rate, but significantly increased upon transfer to the artificial intestinal fluid, with a 163% survival rate (Supplementary Table 1). This indicated that LGG did not react with pepsin and trypsin, was resistant to acid and bile salts, and could pass through the digestive tract with a sufficient number of viable bacteria.

### LGG improves femur microstructure and biomechanics in OVX rats

**Figure 2.**
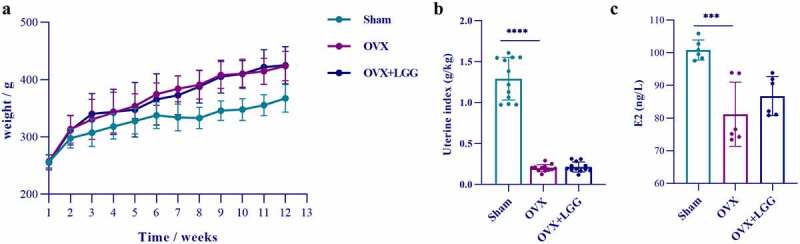
(a) Changes in body weight of experimental animals over the course of the experimental period. (b) Differences in uterine index (uterine weight/body weight) among groups. (c) Serum E2 level.

Removal of bilateral ovaries in female rats leads to estrogen deficiency and uterine atrophy. The uterine index (uterine weight/body weight) of OVX rats was significantly decreased (Figure 2(b)), which also indicated that the model was successful, and the administration of LGG had no effect on uterine atrophy caused by estrogen deficiency. The serum estrogen (E2) levels in the OVX group decreased notably (Figure 2(c)), indicating that estrogen expression was reduced after ovariectomy. LGG administration increased serum E2 levels, but the effect was not significant. The Figure 2(a) shows the comparison of the body weight in each group throughout the experiment, and the trend of weight increased markedly in OVX and LGG groups compared with the sham group. Therefore, LGG treatment had no significant effect on the weight and uterine atrophy induced by estrogen deficiency.

**Figure 3.**
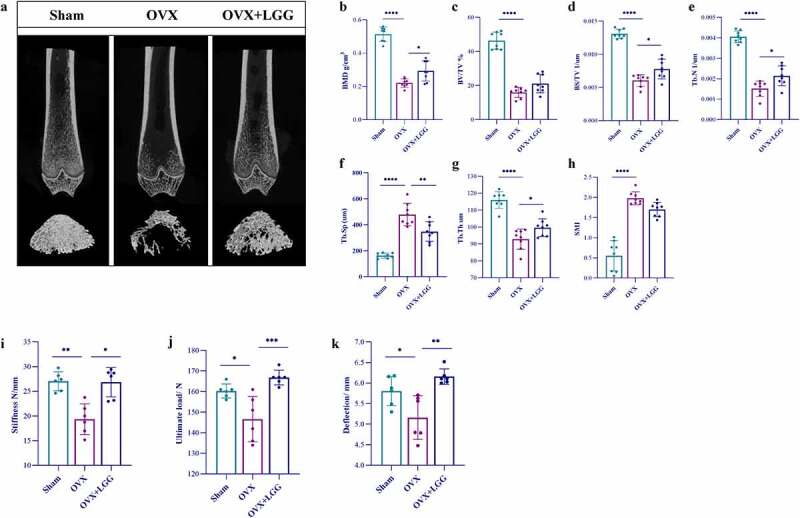
LGG ameliorates osteoporosis in ovariectomized rats. (a) the femur distal metaphyseal region of rats was analyzed using micro-computed tomography (CT). (b-h) Comparison of BMD, BV/TV, BS/TV, Tb.Sp, Tb.N, Tb.Th, SMI between groups. (i-k) Biomechanics of the femur of ovariectomized rats; a three-point bending test was used to detect the stiffness, ultimate load, and deflection. Data are expressed as mean ± standard deviation. (*n* = 6–12). *P < 0.05, **P < 0.01, ***P < 0.001, ****P < 0.0001 (Dunnett multiple comparisons test). BMD, bone mineral density; BV/TV, bone volume/total volume; BS/BV, bone surface area/bone volume; Tb.Sp, trabecular separation; Tb.N, trabecular number; Tb.Th, trabecular thickness; SMI, structure model index; LGG, *Lactobacillus rhamnosus.*

To explore the effect of LGG on estrogen deficiency-induced bone loss, the bone microstructure of the distal femur was evaluated using micro-computed tomography (CT). The microstructure of the bone trabeculae was visualized using three-dimensional (3D) images of the distal femur (Figure 3(a)). Compared to the sham group, the distal femoral trabeculae in the OVX group were thinner, fewer, less dense, sparsely arranged, and had an enlarged bone marrow cavity. LGG treatment ameliorated bone loss and improved the microstructure of the bone trabeculae. Bone surface (BS)/total volume (TV) and bone volume (BV)/total volume (TV) can indirectly reflect the amount of bone volume, and bone metabolism. The number of trabeculae (Tb.N), trabecular thickness (Tb.Th), and trabecular separation (Tb.Sp) were used to evaluate the spatial morphological structure of trabeculae. The structural model index (SMI), describing the ratio of lamellar to rod-like structures in the structural composition of trabecular structures, generally increases in osteoporosis, indicating that the trabeculae shift from lamellar to rod-like. LGG treatment showed an increase in bone mineral density (BMD), BV/TV, BS/TV Tb.N, and Tb.Th, and decrease in Tb.sp to varying degrees and improved the trabecular SMI (Figure 3(b-h)). Overall, LGG treatment ameliorated the microstructural loss in OVX rats.

The biomechanical properties of rat femurs were measured using a three-point bending test. Femur stiffness, ultimate strength, and fracture deflection were significantly decreased in the OVX group and were significantly improved after LGG treatment (Figure 3(i-k)), indicating that probiotic LGG can enhance bone strength in estrogen-deficient rats.

### LGG modulates bone turnover induced by estrogen deficiency

**Figure 4.**
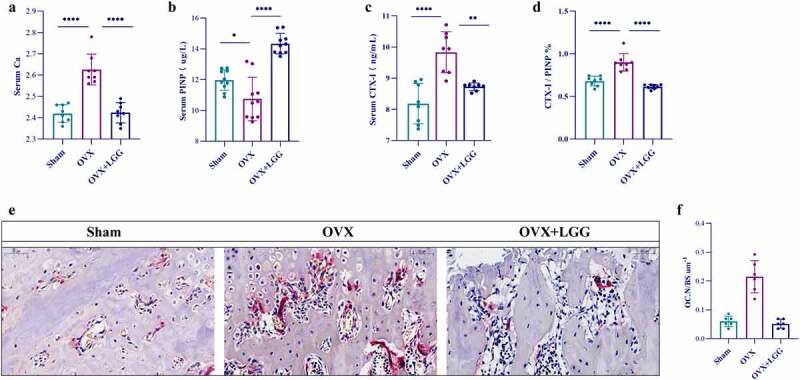
Administration of LGG improves expression of bone turnover markers. (a-d) the changes of serum Ca level, CTX-I, and PINP, and CTX-I/PINP were observed among the groups. (*n* = 8–10). (e) Representative pictures in TRAP stained (scale bar, 100 µm; arrows, red-wine stained osteoclasts with multiple nuclei) (*n* = 3). (f) OC.N/BS of osteoclasts from slices of distal femur was evaluated in each group in two different fields of view. (*n* = 3). Data are expressed as mean ± standard deviation. (*n* = 6–12). *P < 0.05, **P < 0.01, ***P < 0.0001 (Dunnett multiple comparisons test). CTX-I, C-telopeptide of type I collagen; PINP, N-terminal propeptide of type I procollagen; OC.N, the number of osteoclast; OC.N/BS, the number of osteoclast/the bone surface area.

To assess the anti-osteoporotic effect of LGG, changes in the serum levels of bone biochemical markers were examined. Serum calcium levels significantly increased in the OVX group (Figure 4(a)), indicating increased bone resorption after estrogen deficiency, and LGG significantly reduced this expression. PINP expression was decreased in OVX rats compared to sham-operated rats, but there was no significant difference; however, LGG significantly elevated serum PINP levels (Figure 4(b)). Serum CTX-I levels were noticeably higher in the OVX group (Figure 4(c)), indicating a significant increase in bone resorption activity, which was reduced by LGG treatment. The ratio of the bone conversion markers is shown in the Figure 4(d), with a significant increase in CTX-I/PINP in the OVX group and a return to the sham equivalent level after LGG treatment.

The bone turnover index results for bone metabolism implied more significant changes in the osteoclastogenic factor CTX-I in the OVX model. Therefore, the histomorphology of femurs was assessed using tartrate resistant acid phosphatase (TRAP) staining to explore the effect of LGG on estrogen deficiency-induced bone loss. Representative images (Figure 4(e)) depicting osteoclasts as positive cells with more than three nuclei and peripheral staining in red show that estrogen reduction leads to a significant increase in osteoclasts which LGG can counteract. LGG treatment ameliorated bone loss in OVX rats when the number of osteoclast/the bone surface area (OC.N/BS) was selected for measurement (Figure 4(f)).

### LGG strengthens the intestinal barrier

**Figure 5.**
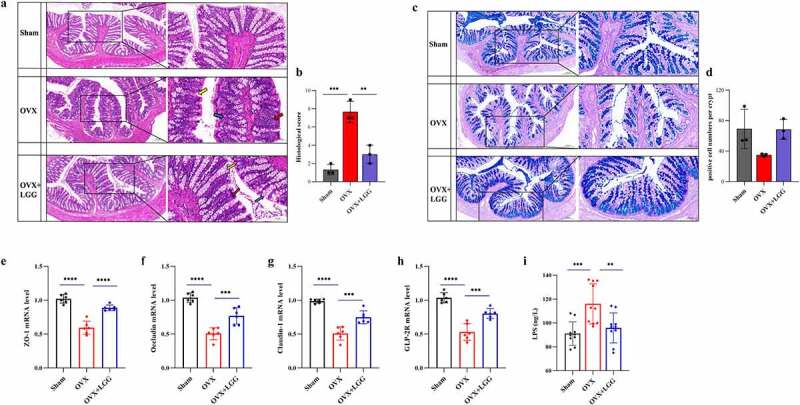
Administration of LGG induces gut barrier reinforcement. (a) Representative pictures of HE-stained (scale bar, 200 µm, enlarged figure, 100 µm (*n* = 3). Red arrows, inflammatory infiltrates; blue arrows, goblet cells; yellow arrows, intestinal crypt). (b) Intestinal morphology studies measure cumulative scores including inflammation infiltration and crypt damage and goblet cells damage.The scoring criteria are shown in Supplementary Table 2. (c) Representative pictures of Ab-PAS stained (scale bar, 200 µm, enlarged figure 100 µm) (*n* = 3). (d) Morphological research measures include the number of goblet cells in the gland. (e-h) the mRNA expression of the ZO-1, occludin, claudin-1, and GLP-2 gene in colon tissue. (*n* = 6). (i) Serum LPS level. (*n* = 10). Data are expressed as mean ± standard deviation. *P < 0.01, **P < 0.001, ***P < 0.0001 (Dunnett multiple comparisons test). HE staining, hematoxylin-eosin staining; Ab-PAS stained, Alcian blue – periodic acid Schiff stain; ZO-1, Zonula Occludens-1; GLP-2, Glucagon-like Peptide-2; LPS, lipopolysaccharide; LGG, *Lactobacillus rhamnosus.*

Increased intestinal permeability plays a key role in the upregulation of inflammation. To assess the effect of LGG on the impaired intestinal barrier induced by estrogen deficiency, the colonic barrier was observed using hematoxylin and eosin (HE) staining analysis of the intestinal structure. The representative figures (Figure 5(a)) show that the mucosal, mucosal muscle, submucosal, and muscle layers of the sham group are obvious and clear with abundant well-arranged intestinal crypts, absence of pathological changes such as inflammation, necrosis, and edema, and normal morphological structure. Ovariectomy causes local damage to the tissue and mucosa, and the intestinal glands are atrophied, deformed, and partially replaced by proliferating fibrous tissue with inflammatory cell infiltration. LGG treatment reduced mucosal damage and inflammatory cell infiltration and improved crypt structure (Figure 5(b)). Mucosal layer thickness, studied using Alcian blue (Ab)-periodic acid – Schiff (PAS) staining, is considered a critical barrier that responds to intestinal barrier function and protects epithelial cells from harmful factors (Figure 5(c)). We found that LGG ameliorated goblet cell atrophy, increased the number of goblet cells in the gland, and promoted mucus production compared with OVX (Figure 5(d)). In addition, we found that the mRNA expression levels of the TJ proteins ZO-1, occludin, and claudin significantly increased after LGG treatment (Figure 5(e-g)). GLP-2 R, mainly present in the gastrointestinal tract, is the major mediating receptor for GLP-2, and its expression was increased after LGG treatment (Figure 5(h)). GLP-2 is considered to maintain the intestinal villus epithelium growth and absorption function, which is closely related to intestinal permeability and systemic inflammatory phenotype, and is an important complement to intervention studies on the effect of prebiotics or probiotics on the intestinal barrier.^23^ LPS is present in the outer membranes of most gram-negative bacteria, and serum LPS is considered to be a potential marker of increased intestinal permeability, and more specifically, of bacterial translocation.^24^ Serum LPS expression decreased significantly after treatment with LGG (Figure 5(i)).

### LGG regulates Th17/Treg and related cytokine levels in mesenteric lymph nodes

**Figure 6.**
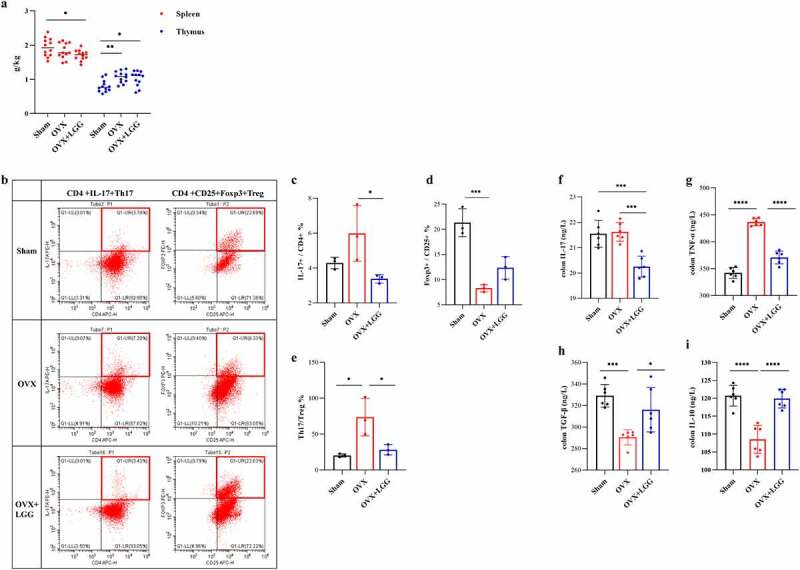
Administration of LGG improves the Th17/Treg balance in gut. (a) Thymus and spleen index (organ weight/body weight). (*n*=(12). Frequencies of Th 17 cells and T reg in MLN. (c-d) Th17(CD4+IL-17A+), Treg(CD4+CD25+FOXP3+). (n = 3). (e) the IL-17+/FOXP3+ % value represents the difference of Th17/Treg ratio between groups. (n = 3). (f-i) Colonic pro-inflammatory cytokines concentration. IL-17, TNF-α; colonic anti-inflammatory cytokine concentration. IL-10, TGF-β. (n = 6). Data are expressed as mean ± standard deviation. *P < 0.05, **P < 0.01, ***P < 0.001, ****P < 0.0001 (Dunnett multiple comparisons test). Th 17, T helper cell 17; T reg, Regulatory T cell; MLN, mesenteric lymph nodes; LGG, *Lactobacillus rhamnosus.*

The thymus index (thymus weight/body weight) was significantly higher in the OVX group, indicating possible organismal inflammation in the OVX group, and LGG was able to reduce it slightly; the spleen index (spleen/body weight) was not significantly different between groups, but decreased with LGG treatment (Figure 6(a)).

We compared the CD4+IL-17A+ Th17 cell population with the CD4+CD25+FOXP3+ Treg cell population in mesenteric lymph nodes (MLN) to confirm the inflammatory response in the intestine (Figure 6(b)). Due to the variability in the size of the tissue taken from the intestine MLN, the calculation of the absolute number of flow cells was likely unreliable. Therefore, the proportional IL-17+/CD4+ percentage was calculated to represent the Th17 cell population and FOXP3+/CD25+ percentage to represent the Treg cell population, further reflecting the inflammatory status of the MLN with IL-17+/FOXP3+ percentage. The number of Th17 cells was increased in the OVX group compared to the sham group and was downregulated with LGG administration while the opposite trend was seen in the case of Treg cells (Figure 6(c,d)). Furthermore, IL-17+/FOXP3+ percentage analysis indicated that intestinal Th17/Treg imbalance occurred in estrogen deficiency-induced osteoporosis, and LGG treatment significantly downregulated this percentage, having a significant effect on intestinal inflammation (Figure 6(e)).

The expression of inflammatory factors in the colon was further examined (Figure 6(f-i)). Colon TNF-α levels were significantly increased in the OVX group compared to the sham group, and LGG treatment significantly reduced the expression. Colon IL-17 expression was not very different between OVX and sham groups, but LGG treatment significantly reduced colon IL-17 expression compared to the other groups. IL-10 and TGF-β are related to Treg cytokines that have anti-inflammatory effects. OVX significantly decreased the expression of IL-10 and TGF-β in the colon compared to that in the sham-operated group, and LGG increased this expression. These results suggest that the effect of LGG treatment on intestinal inflammation caused by estrogen deficiency may be associated with the inhibition of Th17 cell production and expression of related cytokines IL-17 and TNF-α, promotion of MLN Treg cell production, and promotion of the expression of anti-inflammatory cytokines IL-10 and TGF-β.

### LGG regulates Th17/Treg in femurs induced by estrogen deficiency and correlates with colonic inflammation

Considering the extensive study of the gut – bone axis connection as well as the inflammatory response, to the expression of RoRγt and FOXP3 was selected to investigate the Th17/Treg ratio in the BM. Before this, the expression of inflammatory and anti-inflammatory factors in serum was studied (Supplementary Figure 1). TNF-α and IL-17 were significantly increased and IL-10 expression was significantly decreased in the OVX group. LGG treatment decreased TNF-α and IL-17 expression and increased IL-10 and TGF-β expression. The general trend is consistent with the expression of colonic inflammatory factors.

**Figure 7.**
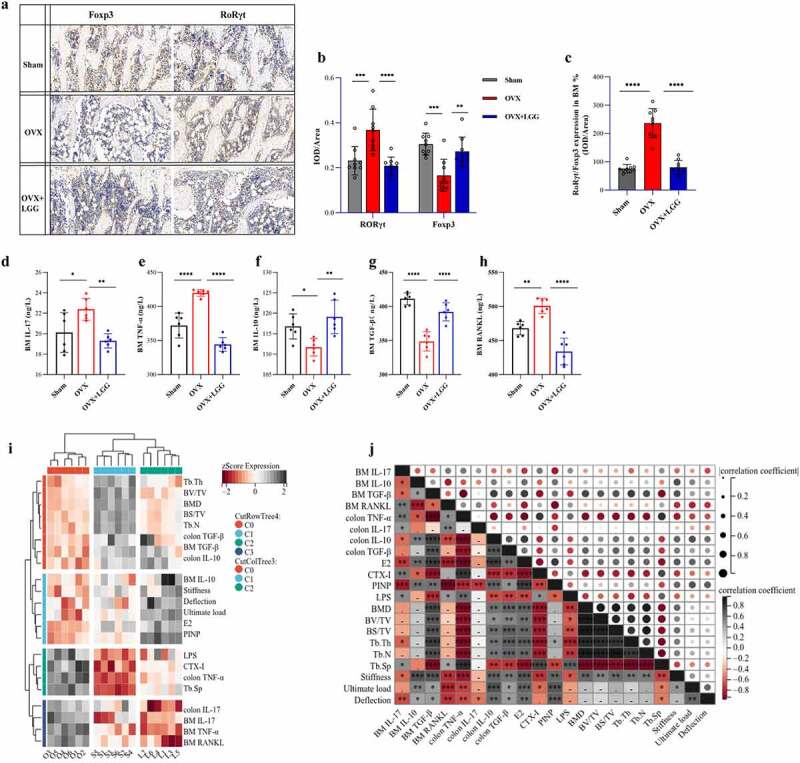
Administration of LGG improves the Th 17/T reg balance in BM. (a) Representative pictures of immunohistochemistry staining of RORγt and FOXP3 in femur tissue of the three groups (scale bar, 100 µm). (b) Measurements included IOD/area analysis, with each sample analyzed in three different fields of view. (*n* = 3). (c) RORγt/FOXP3 was used to measure Th17/Treg in BM. (d-g) Pro-inflammatory cytokines, IL-17 and TNF-α, and anti-inflammatory cytokines concentration, IL-10 and TGF-β, in BM. (*n* = 6). (h) Expression of osteoclast cytokine RANKL in BM. (*n* = 6). (i-j) Analysis of the correlation between the bone and Th17/Treg. Heat map analysis of bone indexes and Th17/Treg, and cluster analysis among samples in groups. Matrix analysis for correlation analysis of all samples. Data are expressed as mean ± standard deviation. *P < 0.05, **P < 0.01, ***P < 0.001, ****P < 0.0001 (Dunnett multiple comparisons test). Th 17, T helper cell 17; T reg, Regulatory T cell; IOD, integral optical density.

Representative figures (Figure 7(a)) show increased expression of RoRγt in OVX compared to sham and effective downregulation by LGG treatment, whereas FOXP3 expression was significantly decreased in OVX and upregulated by LGG treatment. Quantitative analysis was performed using Image Pro Plus, and comparative analysis between groups was performed using the integral optical density (IOD)/area (Figure 7(b)). To better visualize the changes in Th17/Treg cells, we further assessed the trend and ratio of RORγt/FOXP3 in the BM (Figure 7(c)). The expression of pro- and anti-inflammatory cytokines in the BM was further examined (Figure 7(d-g)). TNF-α and IL-17 levels were significantly increased in the OVX group, and LGG treatment decreased their expression. Additionally, the expression of IL-10 and TGF-β was significantly decreased in the OVX group, and LGG increased the expression, which suggests that estrogen deficiency produced Th17/Treg imbalance in the OVX group, and the corresponding expression trends of Th17/Treg in the gut and bone were similar. RANKL is an osteoclast-associated factor and important regulator of osteoclast differentiation. It was significantly higher in the OVX group than in the sham group, and LGG treatment significantly downregulated its expression (Figure 7(h)).

To further visualize the correlation between the inflammatory response generated by the gut and bone tissue and the correlation between osteoporosis and Th17/Treg in OVX, we integrated the data and heat map for correlation analysis. The results were consistent with our expected hypothesis, with significant correlation between osteogenic and anti-inflammatory factors, and between osteoclastic and pro-inflammatory factors, and closer trends between sham and LGG-treated groups (Figure 7(i)). The specific correlations between the impact factors are presented in Figure 7(j)).

### LGG alters intestinal microbe diversity in OVX rats

**Figure 8.**
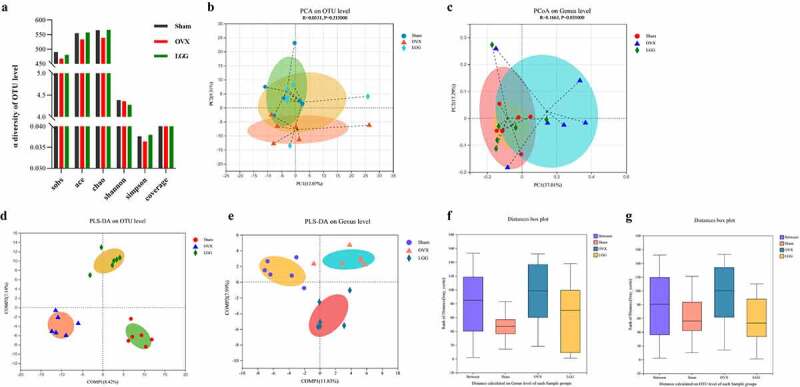
Cluster and diversity analysis of gut microbiota in mice in different groups. (a) Reflecting the alpha diversity of the gut microbes in feces, Sobs, ace, Chao, Shannon, Simpson, and coverage index changes in each group. (b) PCA plot of the gut microbiota at the OTU level. (c) PCoA plot on genus level. (d,e) PLS-DA plots on OTU and genus levels. (f,g) Beta diversity analysis of ANOSIM analysis plot of the gut microbiota in feces on OTU and genus levels. PCA, principal component analysis; OTU, operational taxonomic unit; PCoA, principal coordinate analysis; PLS-DA, partial least-squares discriminant analysis; ANOSIM, analysis of similarities.

Our previous study showed that in an OVX rat model with concomitant gut flora dysbiosis, there were differences in species richness and composition compared to the sham group. To understand whether the effect of LGG on osteoporosis caused by estrogen deficiency is related to the regulation of intestinal flora, we analyzed the composition of intestinal flora by sequencing 16S rRNA gene amplicons in the feces of each group of rats. First, we determined whether the amount of sample data used for sequencing was reasonable and sufficient for diversity analysis. Sobs and Shannon indices tended to flatten the dilution curve, and the amount of sequencing data was sufficient to reflect the vast majority of the microbial diversity in the sample (Supplementary Figure 2). The Sobs, ACE, and Chao indices were used to characterize community species richness, where LGG improved the community richness index. Shannon and Simpson indices characterize community diversity; LGG treatment had little effect on the Shannon index but could improve the Simpson index value compared with that of the OVX group (Figure 8(a)). This indicated that LGG treatment increased the abundance and community diversity of the intestinal flora and improved community richness. The results of principal component analysis (PCA) analysis showed that at the operational taxonomic unit (OTU) level (Figure 8(b)), the OVX and sham groups could be better separated, and LGG group was close to and overlapped with the sham. At the genus level, the principal coordinate analysis (PCoA) results showed that the LGG group highly overlapped with the sham group, further indicating that LGG had a regulatory effect on intestinal flora (Figure 8(c)). Whether the difference between groups was significant or not, we illustrated this with the help of partial least-squares discriminant analysis (PLS-DA) (Figure 8(d,e)), which showed a significant separation between groups at the OTU and genus levels, and analysis of similarities (ANOSIM) analysis further represented a significant difference between groups (Figure 8(f,g)).

### LGG alters the composition of intestinal flora at the multispecies level in OVX rats

**Figure 9.**
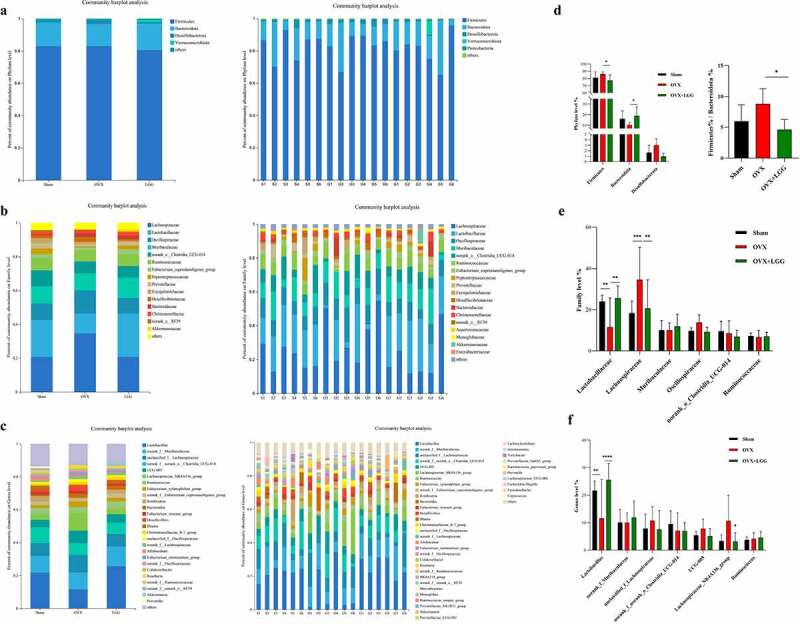
Relative abundance analysis of gut microbiota at the multispecies level in different groups. (a-c) Relative abundances of the gut microbiota at the phylum, family, and genus levels. Each column represents a sample, and each column represents a group. (d-f) the three most abundant phylum, as well as the six most abundant families, and the seven most abundant genera were analyzed separately.

To study the specific changes in bacterial communities, cluster histograms were drawn to show the changes in intestinal flora at the phylum, family, and genus levels in each sample, as well as in the subgroups (Figure 9(a-c)). The relative abundances of the dominant bacterial phyla, families, and genera were also compared between the groups (Figure 9(d-f)).

As shown in the figure, Firmicutes and Bacteroidetes were dominant in all groups at the phylum level, and their percentages were analyzed separately, which showed that the relative abundance of Firmicutes and Desulfobacterota increased and that of Bacteroidetes decreased in the OVX group; LGG treatment reduced the relative abundance of Firmicutes and Desulfobacterota, and increased that of Bacteroidetes. The Firmicutes/Bacteroidetes relative abundance ratio for OVX increased in our study, and LGG was able to decrease it, which was consistent with the sham group (Figure 9(g)).

At the family level, compared to the sham group, OVX reduced the relative abundance of Lactobacillaceae, Ruminococcaceae, Eubacteriumc-oprostanoligenes_group, Peptostreptococcaceae, Prevotellaceae norank_Clostridia_UCG-014, while the relative abundance of Lachnospiraceae, Oscillospiraceae, Desulfovibrionaceae, and Bacteroidaceae significantly increased. LGG treatment restructured the bacterial flora. Compared to OVX, LGG increased the relative abundance of Lactobacillaceae, Ruminococcaceae, Eubacte-rium_coprostanoligenes_group, and Peptostrepto-coccaceae, and decreased the relative abundance of Lachnospiraceae, Oscillospiraceae, and Desulfovibrionaceae, but had little effect on the changes in Prevotellaceae, Bacteroidaceae, and norank_Clostridia_UCG-014, which made the overall flora structure similar to that of the sham group at the family level. Interestingly, we found that the LGG treatment simultaneously increased the relative abundance of Muribaculaceae and Akkermansiaceae, but this did not change significantly in the other groups.

At the genus level, OVX caused the relative abundance of Lactobacillus, norank_f_nora-nk_Clostridia_UCG-014, norank_f_Eubacte-rium_coprostanoligenes_group, Romboutsia, and Blautia to significantly decrease, while unclassified_f_Lachnospiraceae, UCG-005, Lachnospiraceae_NK4A136_group, Eubacterium-xylanophilum_group, Bacteroides Desulfovibrio, unclassified_f_Oscillospiraceae, norank_f_La-chnospiraceae, Eubacterium_ruminantium_group, and Roseburia relative abundance. LGG treatment increased Lactobacillus, norank_f_Euba-cterium_coprostanoligenes_group, and Rom-boutsia, and decreased unclassified_f_Lach-nospiraceae, UCG-005, Lachnospiraceae_NK4A-136_group, Eubacterium_xylanophilum_group, Desulfovibrio, and Roseburia; however, norank_f_norank_Clostridia_UCG-014, Blautia, Bacteroides, unclassified_f_Oscillosp-iraceae, norank_f_Lachnospiraceae, norank_f_Lac-hnospiraceae, and Eubacterium_ruminantium_ group had no significant effect on the changes. Interestingly, LGG treatment increased the relative abundance of Ruminococcus and Akkermansia and decreased the abundance of Allobaculum, norank_f_Oscillospiraceae, and Colidextribacter, whereas these genera did not significantly change in the other groups.

The Kruskal – Wallis H test for multiple group comparisons showed trends in the relative abundance of the flora among the top 20 species at the family level and the top 30 species at the genus level (Supplementary Figure 3). Student’s *t*-test (equal variance) was used to compare the two groups, and the significant differences between the abundances of the top 10 species at the family level and that at the genus level are shown in Supplementary Figure 4.

### LGG adjusts the structure of gut microbes in OVX rats and resembles that of the sham group

**Figure 10.**
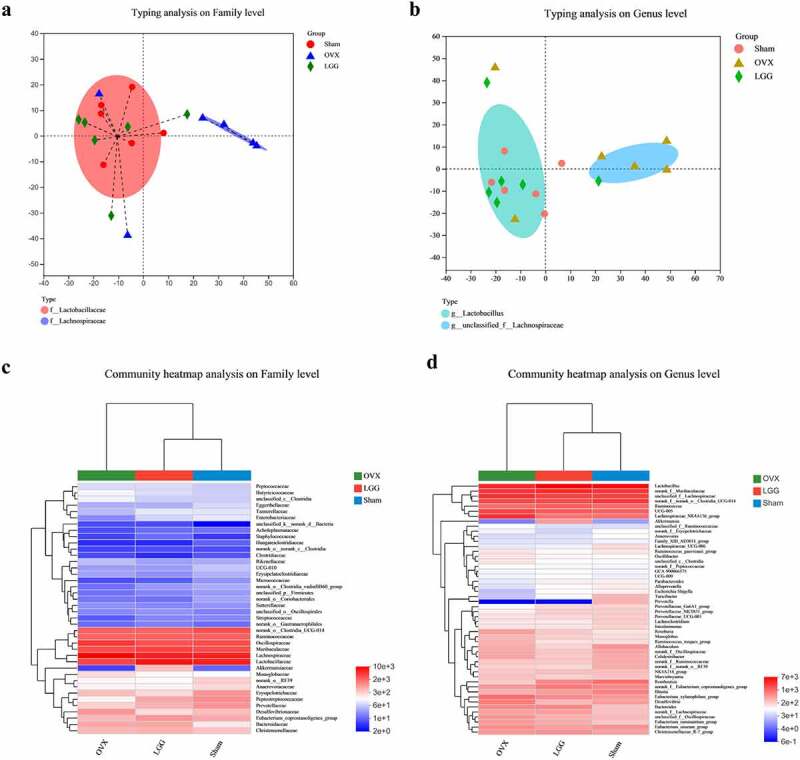
LGG adjusts the structure of the gut microbes of OVX rats. (a,b) Microbiota typing analysis using clustering to study the dominant microbiota structure of different samples at the family and genus levels. (c,d) the heatmaps reflect the similarities and differences in gut microbe community composition of different groups at the family and genus levels.

The Jensen – Shannon Distance (JSD) equidistance was calculated based on the relative abundance of the flora at the family and genus levels, and partitioning around medoids (PAM) clustering was performed. The best clustering K value was calculated using the Calinski-Harabasz (CH) index; the PCoA, K ≥ 2, was used to visualize the samples with similar structures of dominant colonies clustered into one group (Figure 10(a,b)). Sham and LGG were clustered into one class, with the dominant species Lactobacillaceae at the family level and *Lactobacillus* at the genus level. OVX clustered itself into one class, with Lachnospiraceae at the family level and *unclassified_f_Lachnospiraceae* at the genus level. Clustering was performed according to the similarity of species abundance, and high- and low-abundance species were clustered in blocks, reflecting the similarities and differences in community composition in different groups at the family and genus levels (Figure 10(c,d)). LGG and sham groups were clustered into one group and then clustered with OVX. The Circos sample – species relationship figure more specifically and visually reflects the proportion of dominant species composition, and it also reflects the proportional distribution of each dominant species in each group (Supplementary Figure 5). The structural composition and variation in the abundance of each species in the LGG and sham groups were similar at the family and genus levels.

## Gut microbiota and bone turnover indicators and inflammation indicators show correlation in OVX rats

In the previous correlation analysis, osteogenic indicators were positively correlated with anti-inflammatory indicators, and osteolytic indicators were positively correlated with pro-inflammatory indicators, collectively referred to as osteogenic-related factors (OGF), and osteolytic-related factors (OCF), respectively.

**Figure 11.**
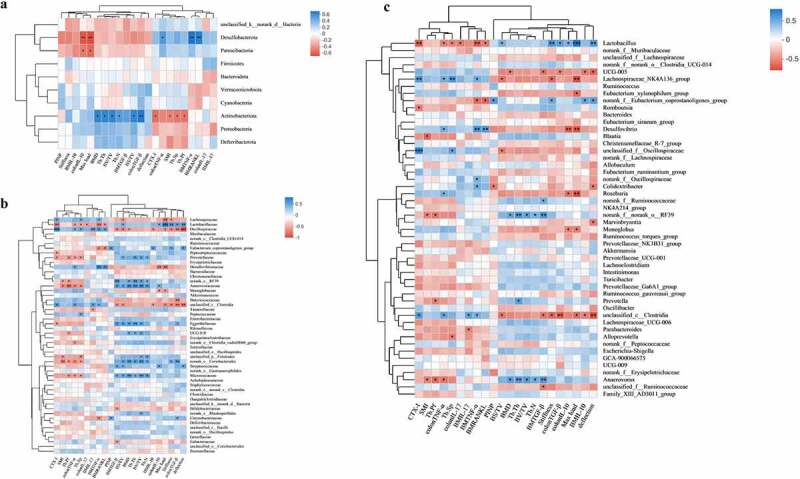
Analysis of the correlation between gut microbiota, bone turnover indicators, and inflammation indicators. (a-c) Heat map of the correlations between 22 OGF and OCF, and gut microbiota at the phylum level, family level, genus level. *P < 0.5, **P < 0.01, ***P < 0.001; non-significant comparisons are indicated by no asterisk. Blue indicates a positive correlation; red indicates a negative correlation. OGF, osteogenic-related factors; OCF, osteoclastic-related factors.

At the phylum level, Desulfobacterota and Patescibacteria were negatively correlated with OGF and positively correlated with OCF; in particular, Desulfobacterota was significantly positively correlated with BM TNF-α, BM RANKL, and colon TNF-α levels, and negatively correlated with colon IL-10 and bone biomechanical index MAx load. In contrast, Actinobacteria and Proteobacteria were positively correlated with OGF and negatively correlated with OCF, and Actinobacteria was significantly negatively correlated with CTX-I and Tp.sp and significantly positively correlated with colon TGF-β, BS/TV, Tb.N, BV/TV, Tb.Th, and BMD (Figure 11(a)).

At the family level, OCF was negatively correlated with Lactobacillaceae, Eubacterium_copro-stanoligenes_group, Prevotellaceae, norank_R-F39, Anaerovoracaceae, Eggerthellaceae, UCG-010, unclassified_p_Firmicutes, norank_Coriob-acteriales, Streptococcaceae, and Micrococcaceae, which were positively correlated with OGF and significantly associated with some indicators. In addition, Lachnospiraceae, Oscillospiraceae, Desulfovibrionaceae, Monoglobaceae, Butyrici-coccaceae, unclassified_c_Clostridia, and Eubacteriaceae were positively correlated with OCF and negatively correlated with OGF, showing significant correlations with some of these indicators (Figure 11(b)). At the genus level, the correlations between flora and OGF and OCF were similar to those at the family level (Figure 11(c)).

**Figure 12.**
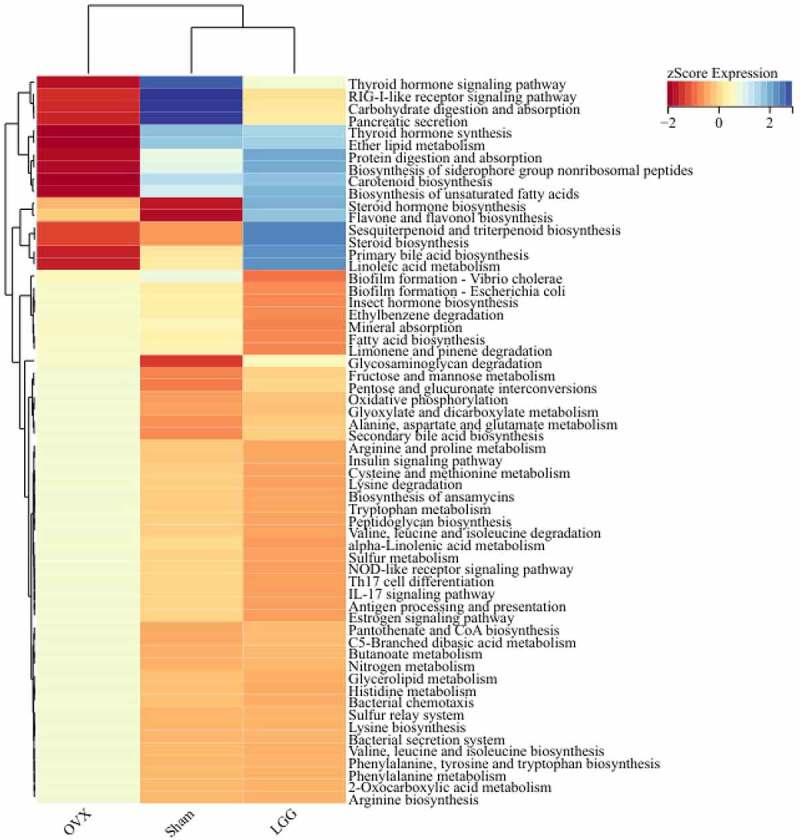
Pathways that are predicted to show different abundances among groups according to the Kyoto Encyclopedia of Genes and Genomes (KEGG) pathway analysis.

The Kyoto Encyclopedia of Genes and Genomes (KEGG) pathway of PICRUSt2 showed differences between the groups in amino acid metabolism, glycan biosynthesis, and metabolism, carbohydrate metabolism, metabolism of terpenoids and polyketides, and lipid metabolism for fatty acid biosynthesis, bile acid biosynthesis, steroid hormone biosynthesis, and biosynthesis of unsaturated fatty acids (Figure 12). In particular, the NOD-like receptor signaling pathway, IL-17 signaling pathways, Th17 cell differentiation, and RIG-I-like receptor signaling pathways in the immune system showed significant differences between OVX and LGG. It has been suggested that LGG might influence these pathways by modulating the microbiome to reduce estrogen deficiency-induced osteoporosis.

### Fecal metabolomics and potential differential metabolites in LGG-treated ovariectomized rats

LGG caused a remodeling of the intestinal flora in ovariectomized rats and the next attempt was to investigate the metabolite due to microbial changes. Therefore, We performed metabolomic analysis of feces from rats after LGG administration (Supplementary Figure 6) to screen for differential metabolites affected by probiotics (Supplementary Figure 7).

Correlation analysis of differential metabolites with inflammatory factors is shown in Supplementary Figure 8. We found that 7-Ethoxy-4-methyl-2 H–1-benzopyran-2-one, Anofinic acid, Bunitrolol, Pindolol, 9-Octadecenamide, Oleamide, 1,3-Diacetoxy-4,6,12-tetradecatriene-8,10-diyne, Myrigalon B, Myrigalone A, Myrigalone E, Verimol C showed a significant positive correlation with the anti-inflammatory cytokines IL-10, TGF-β. Dehydrophytosphingosine, L-Urobilin, 2-(10-Heptadecenyl)-6-hydroxybenzoic acid, 3b-Hydroxy-5-cholenoic acid, 3-oxo-5beta-cholanoic acid, D8’-Merulinic acid C, (3 R, 6‘Z)-3,4-Dihydro-8-hydroxy-3-(6-pentadecenyl)-1 H–2-benzopyran-1-one, Cervonoyl ethanolamide, Cesamet, Nabilone showed a significant positive correlation with the inflammatory cytokines IL-17, TNF-α, RANKL. Because of the correlation between inflammatory factors and bone factors, the above metabolites have similar correlations with osteogenic and osteoclastic factors (supplementary figure 9). Interestingly, we also found that these metabolites which are significantly correlate with immune factors were significantly correlated with the high abundance genera (supplementary figure 10), *Lactobacillus，unclassified_f__Lachnospiraceae，Lachnospiraceae_NK4A136_group，UCG-005，Eubacterium_xylanophilum_group*, the abundance of these genera was retracted after LGG treatment. These metabolites are likely to be key potential metabolites for LGG to play a role in improving osteoporosis in ovariectomized rats.

## Discussion

Many studies have reported the effect and potential mechanisms of probiotics (*Lactobacillus reuteri*,^16^ *Lactobacillus acidophilus*,^25^ *Bacillus clausii* ^17^ on bone homeostasis. Tyagi et al, have well demonstrated that LGG stimulates osteogenesis by producing butyrate to stimulate Treg pools in the intestine and bone marrow in eugonadic young mice,^26^ Sapra et al. further reported the immune potential of LGG to ameliorate osteoporosis in ovariectomized mice in terms of Th17/Treg.^19^ However, the mechanism by which LGG ameliorates osteoporosis through Th17/Treg has not been well reported. The mechanisms of probiotics for metabolic diseases can encompass three main aspects: the modulation of gut microbiota composition, regulation of gut microbial metabolites, as well as improvement of the intestinal barrier function.^27^ We focused on the alteration of the gut microbiome and changes in the gut barrier and associated metabolites by administration of LGG, and from this perspective showed the immune potential of LGG to ameliorate osteoporosis through Th17/Treg immune regulation in ovariectomized rats.

The International Osteoporosis Foundation (IOF) and the International Federation of Clinical Chemistry recommend serum CTX-I (bone resorption) and PINP (bone formation) as two reference markers for osteoporosis.^28^ These were selected as representative markers of osteogenesis and bone resorption, respectively, to reflect bone turnover status in estrogen-deficient rats.^29^ It has also been found that LGG not only decreased CTX-I levels in OVX but increased osteocalcin levels in OVX and Sham which is a bone formation marker.^13^ Similarly, we found that the increased level of CTX-I in the serum of OVX rats was more significant than the decreased level of PINP, and LGG treatment not only reduced CTX-I expression, but also increased the expression of PINP, and it had more pronounced effects on the osteogenic metabolite PINP. Some studies have also shown that LGG also increased bone formation in the sham group, inducing a net bone anabolic effect unrelated to OVX.^13,26^ Combined with our experimental results, this suggests that LGG reduced bone resorption and increased osteogenesis at the same time, and may predominantly promoted osteogenic expression in OVX rats. Our hypothesis suggests that one of the mechanisms involved in LGG treatment for osteoporosis is amelioration of the Th17/Treg imbalance that is caused by the impaired intestinal barrier due to estrogen deficiency. Several studies have shown that the OVX model is accompanied by an impaired intestinal barrier and is strongly associated with the production of inflammation.^13,30^ Our study confirmed this. ZO-1, claudin-1, and occludin are important TJs in which the intestinal barrier exerts a physical defense mechanism.^10^ GLP-2 has been shown to be associated with the intestinal barrier and mucosa in studies of prebiotics and intestinal flora,^31,32^ and administration of prebiotics can directly alter intestinal GLP-2 levels. This was correlated with intestinal flora to show crosstalk between flora and metabolic activity.^33^ Our results showed that LGG administration can significantly improve intestinal barrier damage and the expression of TJs and GLP-2. Here, we suggest that the change in the gut microbiome affects the digestion, absorption, and utilization of ingested food and therefore affects the production of GLP-2 hormone, which in turn has a protective effect on the intestinal barrier to varying degrees. This could be further investigated in bacteria closely related to carbohydrate metabolism, digestion and absorption, and short-chain fatty acid pathways.

In a pathological state, intestinal flora affects the immune system, especially the differentiation of T cells. Sapra et al, showed an increase in the proportion of Th17 and a decrease in the proportion of Treg at different immune sites in ovariectomized mouse, and an increase in the expression of serum inflammatory factors IL-17 and TNF-α associated with osteoclasts, and a decrease in the levels of cytokines such as IL-10 and IL-4. Similarly our study focused on the changes of Th17/Treg in intestine and bone. The proportion of Th17 cell population increased and Treg cells significantly decreased in MLN. Femoral RoRγt and FOXP3 was visualized along with the distribution and expression of Th17 and Treg cells. Consistently, Th17 expression was increased in OVX, Treg expression was decreased, and the trend was alleviated after LGG treatment. And the levels of colonic inflammatory factors TNF-α increased while those of IL-10 and TGF-β decreased in OVX, but there was no significant change in colon IL-17 expression in OVX compared with Sham, which is inconsistent with the change in Th17 predominance detected in MLN. BM inflammatory factors revealed a significant increase in the expression of pro-inflammatory cytokines IL-17 and TNF-α and a significant decrease in IL-10 and TGF-β, which regressed after LGG treatment.

Intestinal T cells are estrogen deficient targets associated with bone loss. In order to further reflect the association of gut-bone axis, we studied the expression of inflammatory cytokines in serum, and the results showed that the expression of pro-inflammatory cytokines IL-17 and TNF-α increased in OVX, while anti-inflammatory cytokines IL-10 decreased. LGG treatment can reduce the expression of pro-inflammatory cytokines and promote the expression of anti-inflammatory cytokines. The general trend is consistent with that in the gut-bone axis, suggesting that inflammatory responses caused by impaired intestinal barrier in OVX may circulate into bone and affect bone homeostasis. Tyagi et al,^26^ found that LGG did not alter the transcript levels of IL-10 in BM, but similarly with the research of Sapra,^19^ we found that the expression of IL-10 increased in BM, colon, serum. Yu et al, had been shown that the immune cell migration in gut-bone axis, and the increase of TNF+ T cells in bone marrow caused by ovarian resection are secondary to the homing of intestinal TNF+ T cells to bone marrow^34^. Therefore, the expression and release of relevant inflammatory factors in these immune cells need to be further studied.

The intestinal tract hosts a large number of microbiota, and disruption of their spatial organization is often a common underlying feature of disease pathogenesis.^10,35^ Such disruption is commonly seen in basic studies of estrogen deficiency-induced osteoporotic diseases, which has led to the study of the gut – bone axis. Regulating the composition of the microbiome is one of the the mechanisms of probiotics for metabolic diseases. In regulating the composition of the microbiome, probiotics directly alleviate disease mainly by regulating the abundance of beneficial and harmful bacteria.^36^ Our study investigates the mechanisms of the gut microbiome in ovariectomized rats after LGG administration in three aspects: intestinal microbe diversity, composition of intestinal flora, the structure of gut microbes. Our study showed changes in the diversity and species richness of the intestinal flora in the OVX model, with Firmicutes, Bacteroidetes, and Desulfobacterota were the dominant phyla, while Desulfobacterota proved to be a harmful group associated with inflammation.^37^ Notably, the Firmicutes/Bacteroidetes ratio increased in the OVX group, which is consistent with previous findings and had been shown in other OVX model studies.^38^ Interestingly, *Akkermansia* increased in abundance after LGG treatment, although it was not the dominant genus and accounted for a smaller percentage in some samples. Recent studies have shown the protective effect of *Akkermansia* in the intestinal barrier and mucus layer,^39,40^ as well as the ameliorative effect on intestinal inflammation.^41^ Furthermore, our results showed that LGG treatment not only modulated the changes in many genera in OVX, but increased the abundance of genera that did not change significantly in other groups, such as the genus *Ruminococcus*, which was shown to be associated with reduced intestinal inflammation and production of secondary bile acids.^42^ LGG also reduced genera that did not change significantly in other groups, such as *Allobaculum*, which suggested have an association with mucus layer degradation and inflammation in recent studies.^43^ *Norank_f_Oscillospiraceae* and *Colidextribacter* were significantly correlated with OGF and OCF. This suggests that LGG treatment can regulate the abundance of other flora and the composition of intestinal flora to perform different functions.

Fecal metabolomic results showed that LGG treatment significantly altered the fecal metabolism of OVX rats. Interestingly, differential metabolites significantly associated with inflammatory and bone factors were also strongly correlated with higher abundance genera, which may be key potential metabolites for LGG in the treatment of osteoporosis. Dehydrophytosphingosine is reported to be a metabolite closely associated with intestinal barrier and immune function.^44^ L-Urobilin is a microbial metabolite associated with intestinal permeability and energy metabolism.^45^ 3b-Hydroxy-5-cholenoic acid and 3-Oxo-5beta-cholanoic acid are bile acid metabolites that are closely associated with intestinal microbes,^46^ and the interaction of bile acids with intestinal flora can exert many metabolic and immunological effects.^47^ However, more research is needed on how these metabolites affect osteoporosis and how they relate to probiotics.

In conclusion, our study elucidates the role of LGG in the treatment of estrogen deficiency-induced osteoporosis. An impaired intestinal barrier and increased intestinal permeability could increase gut – bone Th17/Treg and associated inflammatory cytokine expression, which contributes to the imbalance in bone generation and resorption, resulting in osteoporosis. LGG treatment adjusted the flora composition, restored the impaired intestinal barrier, modified the inflammatory expression of the gut – bone axis, and ameliorated osteoporosis. In particular, we found that LGG treatment seemed to have a greater effect on osteogenesis, as illustrated by similar research. LGG not only regressed the imbalanced abundance of OVX flora, but also adjusted the flora structure, specifically increasing or decreasing the genera associated with intestinal inflammation, endocrine metabolism, and the intestinal mucosal layer, which did not change in other groups. In addition, although the therapeutic research on probiotics is attractive and extensive, most therapeutic approaches and mechanism studies have been validated in animal models, and there is the problem that individual differences in intestinal flora have an impact on clinical trials. Therefore, more basic research is needed to summarize and validate clinical trials with intestinal flora as a therapeutic target.

## Methods

### Preparation and activity characterization of LGG

The frozen tubes of LGG (ATCC7469) (LeZhen Biological, China) lyophilized powder was dissolved in compound solution in a sterile environment, preparing the bacterial suspension, and incubated at 30–35°C for 24–48 h. The obtained strain culture was transferred to MRS medium and incubated at 37°C, and counted by plate counting method to make up to 10^9^ colony forming units (CFU) and stored at 4°C.

ATCC7469 was identified by 16S rDNA species as *Lactobacillus rhamnosus* strain NBRC 3425 16S ribosomal RNA partial sequence.

The bacterial suspensions were inoculated with 1% inoculum in the MRS medium. The absorbance of OD600 was measured at different time points within 35 h. The growth curve of LGG was plotted. The bacterial suspension was inoculated in conditioned medium with pH of 2, 4, 5, and 7, and 0(control), 0.05%, 0.1%, 0.2%, 0.3%, 0.5%, 0.6%, and 0.8% sterile bile salt medium and incubated at 37°C. The OD600 ratio for each culture time was calculated using the medium as the control. LGG bacteria were incubated in artificial gastric juice (hydrochloric acid, pepsin, pH 3), and transferred to artificial intestinal juice (potassium phosphate, sodium hydroxide, trypsin, pH 6.8) for 2 h at 37°C, and incubated for 5 and 7 h at 37°C. Viable cell counts were calculated using the gradient dilution plate counting method.

Final colonization success was obtained by sham with LGG group 16S rRNA sequencing results.

### Preparation and treatment of an ovariectomized model causing osteoporosis

Thirty-six female Sprague-Dawley rats, 3 months old (220–260 g) were provided by the Experimental Animal Center of Hangzhou Medical College (Certificate of Conformity No. SCXK (Zhejiang) 2019–0002). Before the experiments, the rats were placed in an environmental control room with specific pathogen free (SPF)-level constant temperature (20–24°C) and humidity (45–60%) and acclimatized for 7 days with a standard 12 h light/dark cycle. This study was approved by the Animal Ethics Committee of the Nanjing University of Traditional Chinese Medicine (approval number: 202109A025).

The experiments were performed in an SPF barrier environment, and all the experimental materials were UV-sterilized. Experimental rats were anesthetized by intraperitoneal injection of sodium pentobarbital (30 mg/kg; 0.1 mL/100 g). At the intersection of the horizontal line of the femur and spine on both sides, bilateral ovarian tissues were found and removed. In the sham-operated group, the same amount of adipose tissue was removed. After surgery, the legs were injected intramuscularly with penicillin and the wounds were coated with erythromycin ointment.

Postoperative recovery was allowed for 6 weeks. The concentration of bacteria to be administered per day was aspirated, the sample was centrifuged. The supernatant was discarded and the pelleted cells were mixed with sterile saline to adjust the concentration of LGG to 10^9^ CFU/mL. The cells were administered at 10^9^ CFU/d by gavage to each rat, and an equal volume of saline (1 mL/d) was administered to the sham-operated and model groups The treatment was continued for six weeks. Body weight was recorded weekly.

### Organs and serum collection

At the end of the experiment, rats were fasted for 12 h and then anesthetized with sodium pentobarbital (30 mg/kg; 0.1 mL/100 g). The rats were sacrificed after blood was collected from aorta abdominalis. Serum was obtained by centrifuging the blood for 10 min at 4000 rpm/min (4°C). The right femur was dissected, removed, fixed in 10% neutral buffered formalin. Part of the right femur was cut off, and about 2 ml of precooled PBS buffer was absorbed with a syringe to flush the bone marrow cavity repeatedly until the cavity turned white. The left femur was removed, one part of which was wrapped in wet gauze for subsequent biomechanical measurements and the other part for subsequent micro-CT analysis. The uterus, spleen, and thymus were weighed and stored at −80°C. Colons were removed about 2 cm and washed with PBS. One part of colon was frozen at −80°C and the rest was fixed in 10% neutral buffered formalin. Mesenteric lymph nodes, 100 mg, were stored in precooled PBS for subsequent analysis.

### Bone histomorphometry study – TRAP staining

The femoral tissues were fixed in 4% paraformaldehyde for 1 week and then placed in EDTA decalcification solution for a further 4 weeks. Paraffin-embedded slices were sectioned to a thickness of 4 μm and TRAP staining was performed (SaiFang Biological, China). Microscopic examination and image acquisition were performed, and the subchondral bone adjacent to the cartilage border was selected as the area defined for observation.

### Biochemical analysis

Serum Ca content was determined by a calcium assay kit (KINGSBIO, China) as well as automatic biochemical analysis (AU 480, Beckman, America). The expression levels of rat estradiol (E2), rat type I procollagen amino-terminal pro-peptide (PINP), and rat type I collagen C-terminal peptide (CTX-I) were measured using ELISA.

The expression of TNF-α, IL-17, TGF-β, IL-10, and LPS was detected using the ground colonic supernatant. TNF-α, IL-17, TGF-β, IL-10, and RANKL were detected in the bone marrow tissue and serum using a commercial ELISA kit according to the manufacturer’s instructions. All ELISA kits were obtained from AiFang Biological (Beijing, China).

### Bone biomechanics – three-point bending test

The surface moisturizing gauze for the femur was removed and the center of the femoral backbone was determined and aligned with the central force loading. The femoral tissue was fixed using a biomechanical tester (Acumen3, MTS, America), the span distance adjusted to 1.2 mm, and loading speed to 0.01 mm/s. The load-displacement curves were plotted, and the stiffness, ultimate strength, and fracture deflection parameters were calculated.

### Bone microstructure detection - Micro-CT

Fixed scans of the isolated distal femur were performed using micro-CT (Skyscan 1176, Bruker, Belgium) under the following conditions: voltage 65 kV, current 385 μA, layer thickness 18 μm, rotation angle 0.24°/r. The growth plate was selected as a reference, and the grayscale range was set to 100 offset, and 50 consecutive images of CT scans were used for bone trabecular region selection. Image reconstruction was performed using the data viewer, CTAn, for region of interest selection, and quantitative data for the trabeculae. In addition, 3D images were obtained using the CTvol and CTvox software (Bruker microCT). Measurements were obtained, including BMD, BS/TV, BV/TV, Tb.N, Tb.Sp, Tb.Th, and SMI.

### Histological evaluation of the colon

Paraffin-embedded colonic tissues were sectioned (4 μm thickness) and stained with HE and Ab-PAS (AiFang Biological). Histopathology was scored as follows: crypt damage (grade 0–4), severity of inflammation (grade 0–3) and cupped cell damage (grade 0–3). Image-Pro Plus (Media Cybernetics, Inc.) was used to observe and count mucus-producing goblet cells in the crypts. Six views were randomly selected from each section.

### Flow cytometry analysis

Approximately 100 mg of mesenteric lymph node tissue was harvested and cut into 2 mm^3^ pieces. The suspension was collected by grinding with a tissue homogenizer, filtered through a 200-mesh screen, and centrifuged at 800 rpm for 10 min. The tissue suspension was incubated with 3 μM of protein transport inhibitor (Beyotime, S1753). Antibodies were added to the tissue suspensions for cell surface staining, APC-anti-mouse-CD4 antibody (Elabscience, 213644; 5 µg/test), PE-cy5-anti-mouse-CD4 (ebioscience,15-0041-81; 0.06 µL/test), and APC-anti-mouse-CD25 antibody (Elabscience 208614; 5 µL/test), and incubated at 37°C for 30 min. Next, 500 μL of fixative (2330512; Invitrogen) was added, incubated for 30 min, and centrifuged at 800 rpm for 10 min to remove the supernatant. Next, 500 μL of membrane-breaking solution (Invitrogen, 2330512) was added and incubated for 30 min. The supernatant was centrifuged, 100 μL of PBS was added, and PE-anti-mouse-IL-17A antibody (Elabscience, 212368 5 μg/Test) or PE-anti-mouse-FOXP3 antibody (Elabscience, 213511 5 μL/Test) was added and incubated at 37°C for 30 min in the dark.

Flow cytometry (BECKMAN COULTER CytoFLEX) was used to detect cell phenotypes, and FlowJo software (Tree Star Inc., San Carlos, CA) was used for analysis. Th17 cells were identified as CD4+IL-17A+ and Treg cells as CD4+CD25+FOXP3+.

### Immunohistochemistry

The fixed rat femur tissues were decalcified, paraffin-embedded, and sectioned at a thickness of 4 μm. Paraffin-embedded tissue sections were immunohistochemically stained with FOXP3 antibody (AiFang Biological) and RORγt antibody (AiFang Biological). Quantification was performed using Image-Pro Plus (Media Cybernetics, Inc).

### RNA extraction and quantitative real-time PCR

Colonic RNA was extracted using the TRIzol total RNA extraction reagent (TIANGEN, DP405–02). The concentration and purity of RNA were determined using the UV absorption method (Thermo Fisher Scientific, NanoDrop® ND-2000). cDNA was synthesized using a reverse transcription kit (RR047B; TaKaRa). qRT-PCR was performed using SYBR Premix Ex Taq (TaKaRa, RR820A) in a real-time PCR system (Applied Biosystems, ABI 7500) using the cDNA. Sense and antisense primers were used for the detection. ZO-1, Occludin, Claudin-1, and GLP-2 primers are listed in Table 1.

**Table 1.** Primers used in RT-PCR analysis.

	Forward(5’−3’)	Reverse(5’−3’)
ZO-1	GGGGAAACCCGAAACTGATGC	GTGGAGAGAAGAGTTGGACAGAGGC
Occludin	GGGACAGAGCCTATGGAACG	CCAAGGAAGCGATGAAGCAA
Claudin-1	ATTTCAGGTCTGGCGACATTAGTGG	ACAGGAGCAGGAAAGTAGGGCAC
GLP-2R	TCCGTCTCCTGTCGCTCCG	AAGGCCCAACCCACCACC
GAPDH	CGTATCGGACGCCTGGTT	AGGTCAATGAAGGGGTCGTT

All primers were synthesized by Sangon Biotech (Shanghai) Co. Ltd. (Shanghai, China).

### DNA extraction and PCR amplification

Total microbial genomic DNA was extracted from fecal samples using the E.Z.N.A.® soil DNA Kit (Omega Bio-tek, Norcross, GA, U.S.) according to the manufacturer’s instructions. The quality and concentration of DNA were determined using 1.0% agarose gel electrophoresis and a NanoDrop® ND-2000 spectrophotometer (Thermo Fisher Scientific) and stored at − 80°C. The hypervariable region V3-V4 of the bacterial 16S rRNA gene were amplified with primer pairs 338F (5’-ACTCCTACGGGAGGCAGCAG-3’) and 806 R (5’-GGACTACHVGGGTWTCTAAT-3’) using a PCR thermocycler (Applied Biosystems). The PCR product was extracted from a 2% agarose gel, purified using the AxyPrep DNA Gel Extraction Kit (Axygen Biosciences, Union City, CA, USA) according to the manufacturer’s instructions, and quantified using a Quantus™ Fluorometer (Promega, USA).

Purified amplicons were pooled in equimolar amounts and paired-end sequenced on an Illumina MiSeq PE300 platform (Illumina, San Diego, USA) according to the standard protocols of Majorbio Bio-Pharm Technology Co. Ltd. (Shanghai, China).

### Fecal metabolomics analysis and metabolites identification

The collected stool samples were removed from the refrigerator at −80°C and thawed at 4°C. Each stool sample (100 mg) was homogenized by adding 1 mL of methanol for 3 min, to precipitate the proteins, and then centrifuged twice (13,000 rpm, 10 min). The supernatant (100 μL) was vortexed by adding 700 uL of methanol, and 20 uL was used for UPLC-Q/TOF-MS analysis.

The analysis conditions refer to our previous study.^20^

The raw data collected via UPLC-Q/TOF-MS were used to align and normalize the chromatographic peaks using the MarkViewTM software. The retention time range was 1–32 min, the mass scan range was 100–1000 Da, the mass tolerance range was 0.05 Da, and the peak intensity threshold was 100. The extracted ion intensities were normalized using the total peak area method. Standard data were imported into SIMICA14.0 for further mathematical model analysis. The partial least squares and orthogonal partial least squares discriminants were used to analyze the dispersion degree of the data and obtain the variable importance in projection (VIP) value. Further import of data into the website of MetaboAnalyst5.0 (https://www.metaboanalyst.ca/) for differential metabolite screening. The metabolites with VIP>1，log2(FC)>2，p < 0.05 were defined as differential metabolites. Identification of differential metabolites by HMDB database (https://hmdb.ca/spectra/ms/search) and PeakView software.

### Data processing and analysis

Bioinformatic analysis of the gut microbiota was conducted using the Majorbio Cloud platform (https://cloud.majorbio.com). Based on the OTU information, rarefaction curves and alpha diversity indices, including observed OTUs, Sobs index, Chao1 richness, ACE index, Shannon index, and Good’s coverage, were calculated using Mothur v1.30.1. Similarities among the microbial communities in different samples were determined by PCoA and PCA based on Bray-Curtis dissimilarity using the Vegan v2.5–3 package. Other analyses were conducted on the (https://cloud.majorbio.com) platform, and the analysis software is shown in Table 2.

RDP Classifier Version 2.2 was used for each OTU representative sequence and 16S rRNA gene database, with a confidence threshold of 0.7. PICRUSt2 (Phylogenetic Investigation of Communities by Reconstruction of Unobserved States) was used to predict metagenomic function based on OTU representative sequences. KO, pathway, and enzyme (EC) information was obtained from the KEGG pathway database, and the abundance of each functional category was calculated based on OTU abundance.

### Statistical analysis

Unless otherwise stated, all data are expressed as the mean ± standard deviation. T-tests were used for comparisons between the two groups, and Dunnett’s multiple comparisons test when comparing multiple groups. Differences were considered to be significant when *P* < 0.05. All statistical analyses were performed using GraphPad Prism 8.2.1 (GraphPad Software Inc.), correlation analysis of the heat map at http://vip.sangerbox.com/and intestinal flora data analysis and drawing on http://login.majorbio.com/.

## Supplementary Material

Supplemental Material

## Data Availability

The data that support the findings of this study will be available in Figshare at DOI: 10.6084/m9.figshare.21293769.
