# Supplementary material for: Lactobacillus rhamnosus GG ameliorates osteoporosis in ovariectomized rats by regulating the Th17/Treg balance and gut microbiota structure
Source: Gut Microbes. 2023 Mar 20;15(1):2190304. doi: 10.1080/19490976.2023.2190304 (PMC10038048; doi:10.1080/19490976.2023.2190304)
Supplement: Supplemental Material [file KGMI_A_2190304_SM4993.docx]

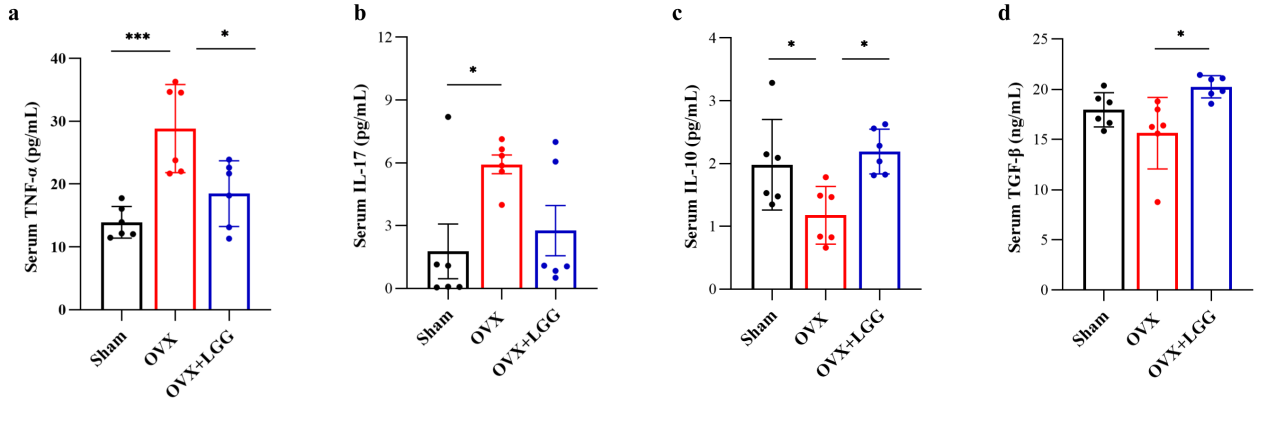


**Supplementary Figure 1.** LGG regulates serum inflammatory factor expression. (a-b) Serum pro-inflammatory cytokines concentration. IL-17, TNF-α; colonic anti-inflammatory cytokine concentration. IL-10, TGF-β. (n=6). Data are expressed as mean ± standard deviation. *P < 0.05, **P < 0.01, ***P < 0.001, ****P < 0.0001 (Dunnett multiple comparisons test).


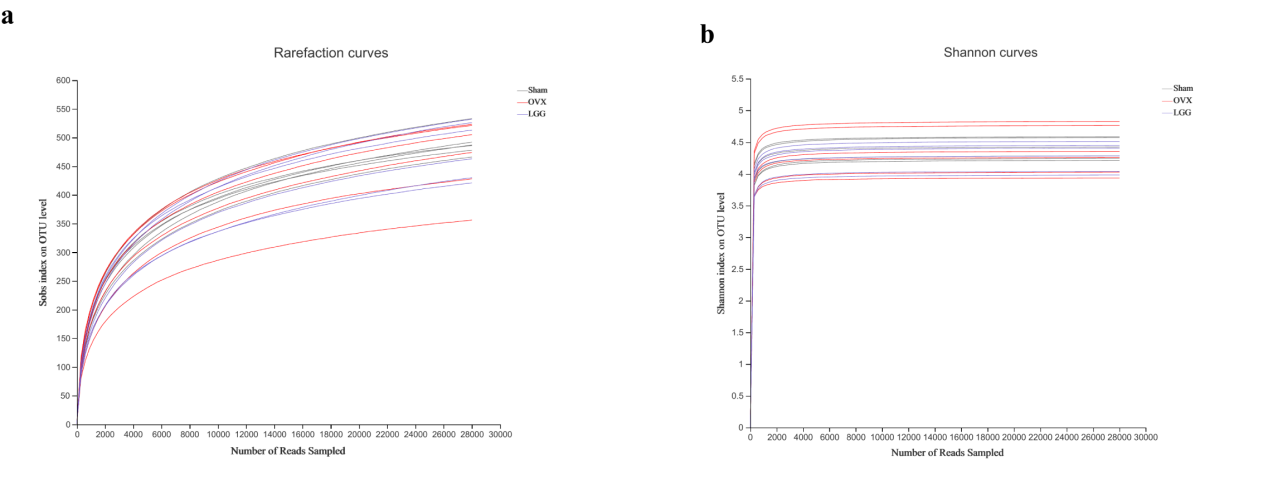


**Supplementary Figure 2.** Rarefaction curves of Sobs (a) and Shannon (b).


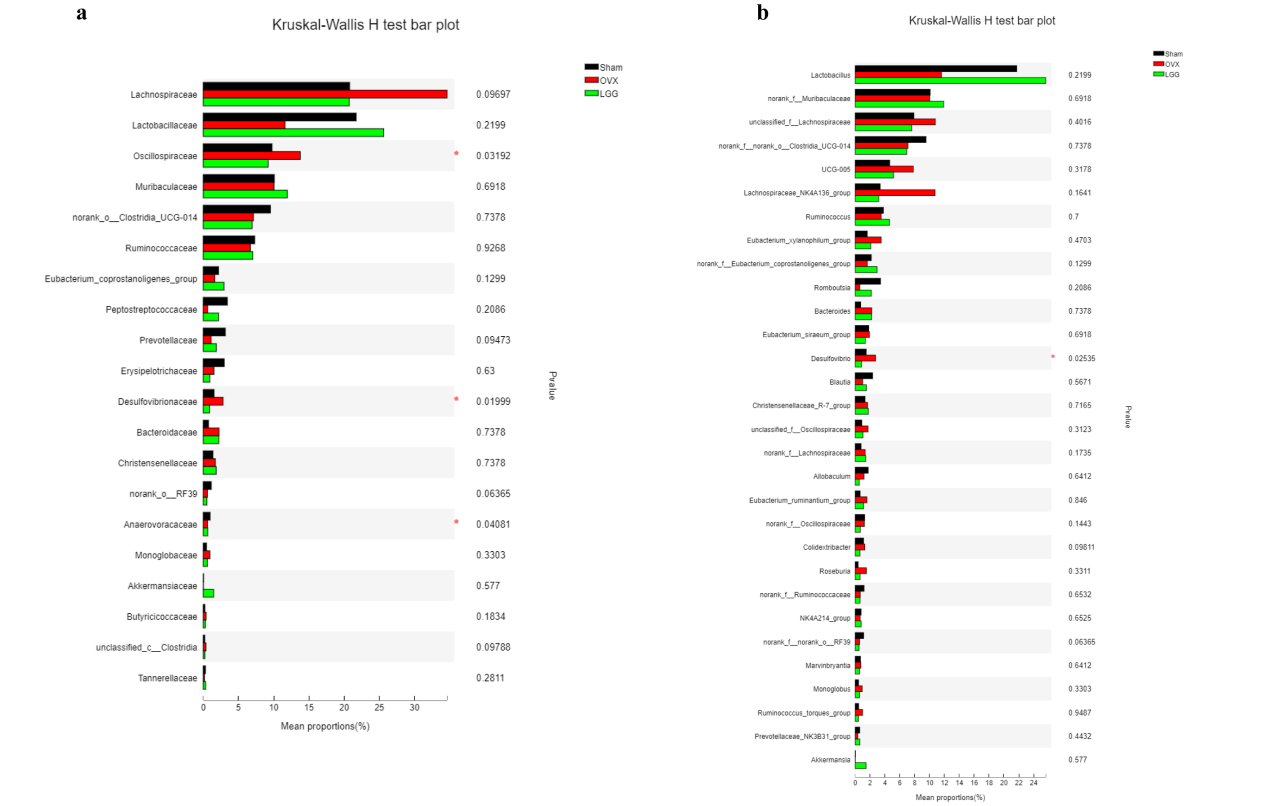


**Supplementary Figure 3.** The Kruskal–Wallis H test for multiple group comparisons. (a) The top 20 species at the family level, (b)The top 30 species at the genus level. The Y-axis indicates the species name at a given taxonomic level, and the X-axis indicates the mean relative abundance in different groupings of species, with different colored bars indicating different groupings; the rightmost is the P-value, *P < 0.05.


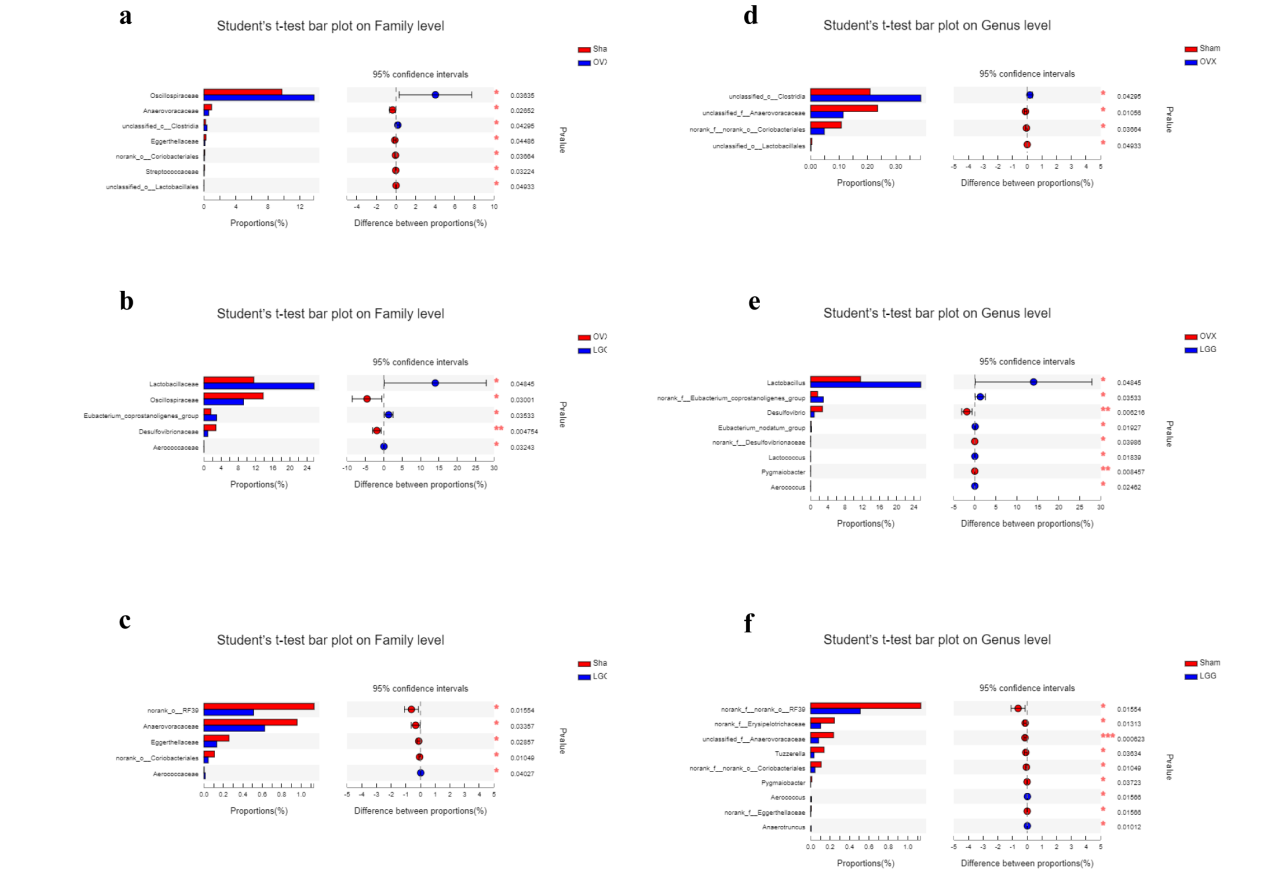


**Supplementary Figure 4.** Student's *t* test for two groups comparisons. The top 10 species abundances at the family(a-c) and genus(d-e) levels. The X-axis represents different subgroups, with different colored boxes indicating different subgroups, and the Y-axis represents the average relative abundance of a species in different subgroups. *P < 0.05, **P < 0.01, ***P < 0.001 .


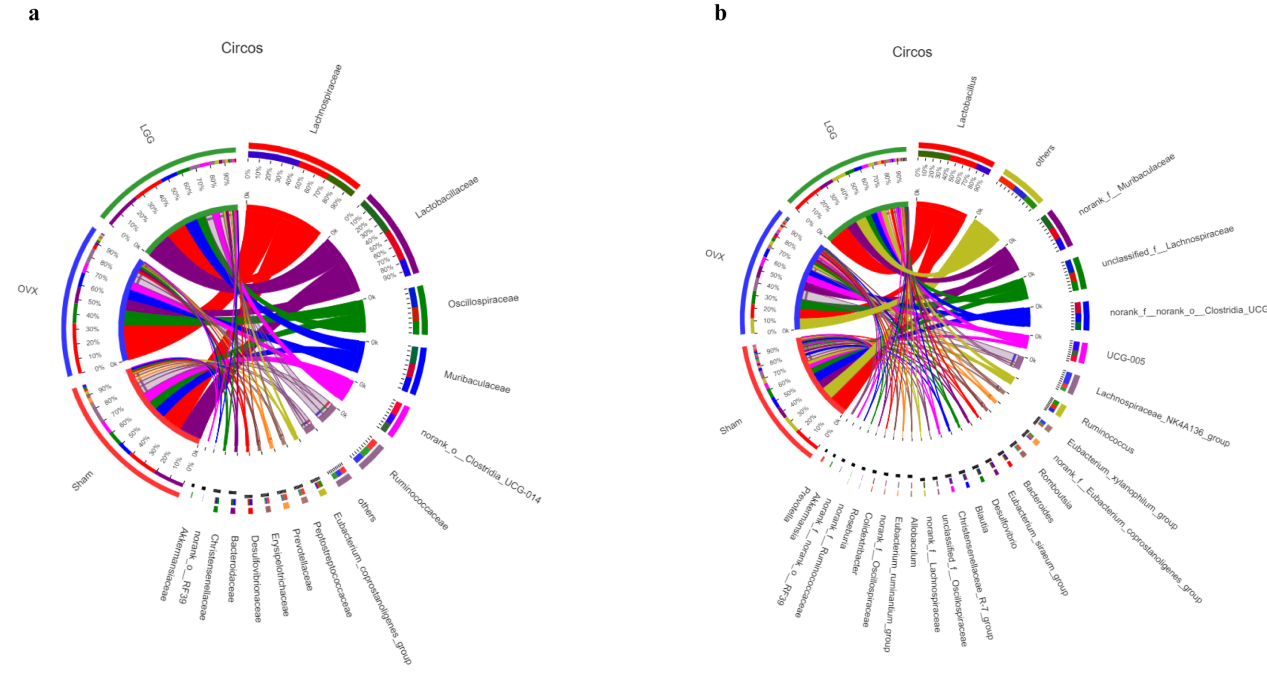


**Supplementary Figure 5.** Circos sample and species relationship diagram. Reflecting the visual diagram of the corresponding distribution proportion relationship between each group and species of family(a) and genus(b).


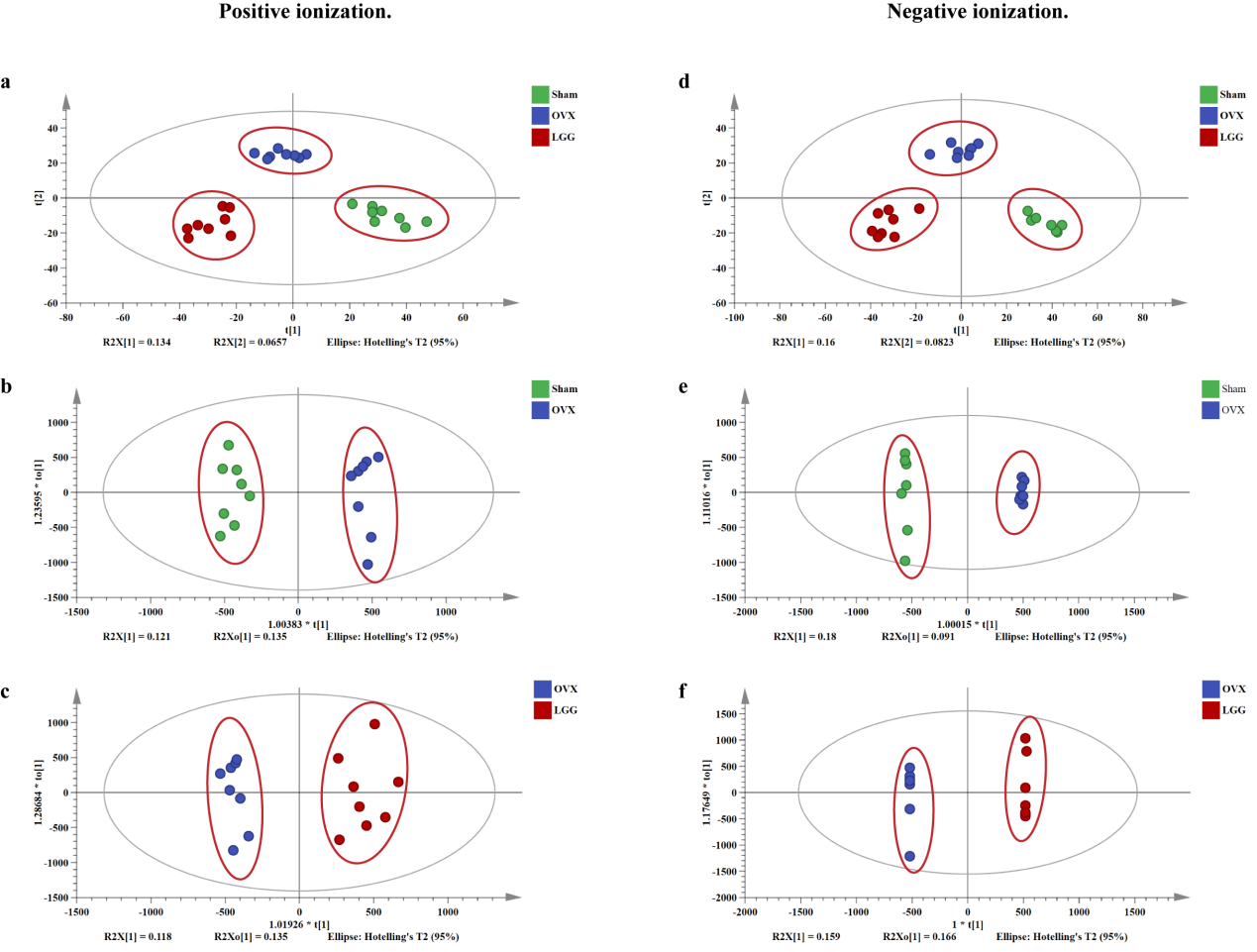


**Supplementary Figure 6.** Metabolomics analysis of fecal samples. (a,d) Partial least squares discriminant analysis (PLS-DA). (b,c,e,f) Orthogonal partial least squares discriminant analysis (OPLS-DA) of positive and negative ionization dataset for rats.


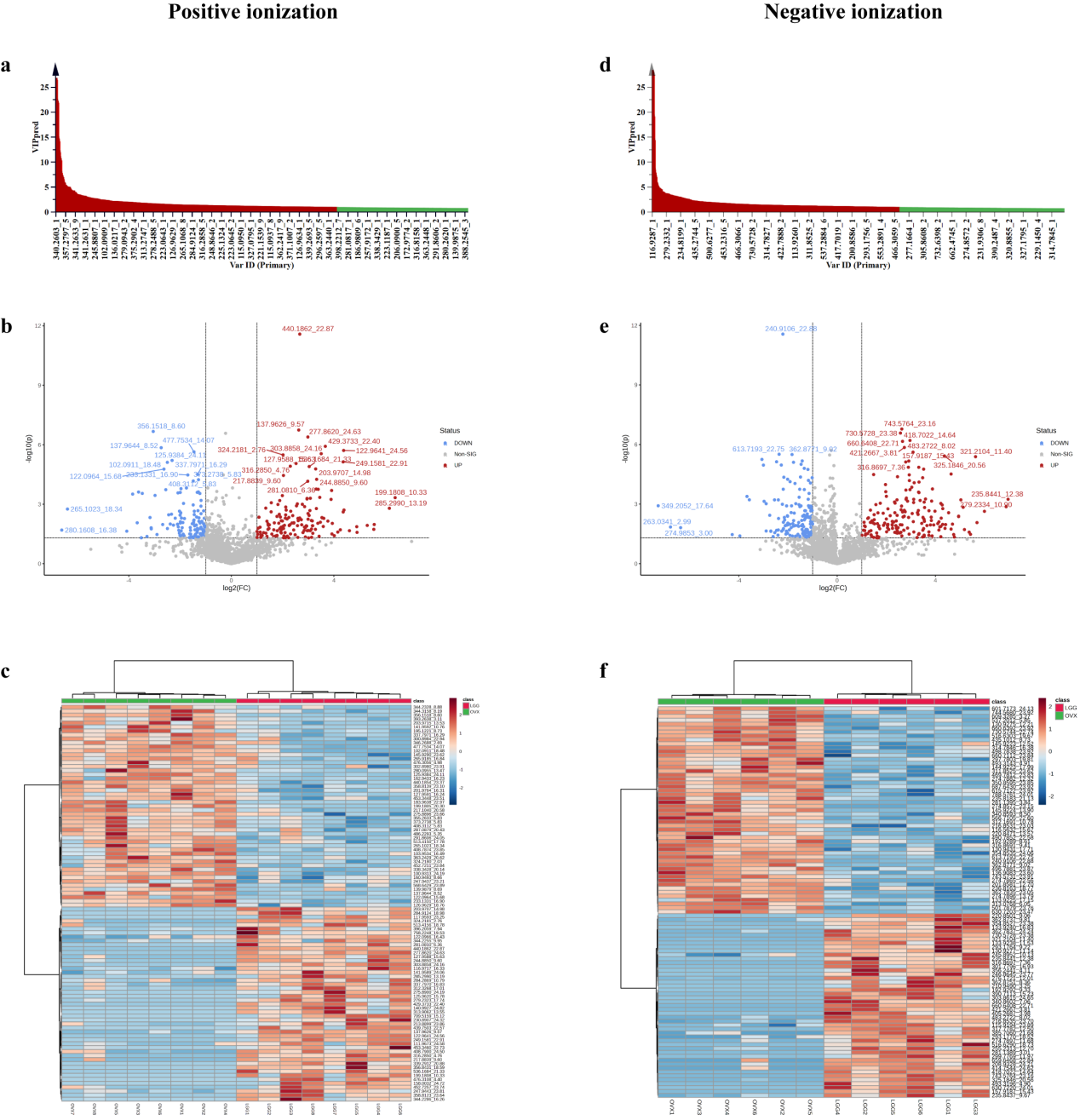


**Supplementary Figure 7.** Differential metabolite profiles of fecal samples. (a,d) Substances with VIP values greater than 1 are meaningful for distinguishing between the two metabolic groups, with red-labeled compounds. (b,e) Volcanic map of all differential metabolites and known metabolites in positive and negative ionization models. (e,f) Heatmap analysis of the fecal metabolite patterns.


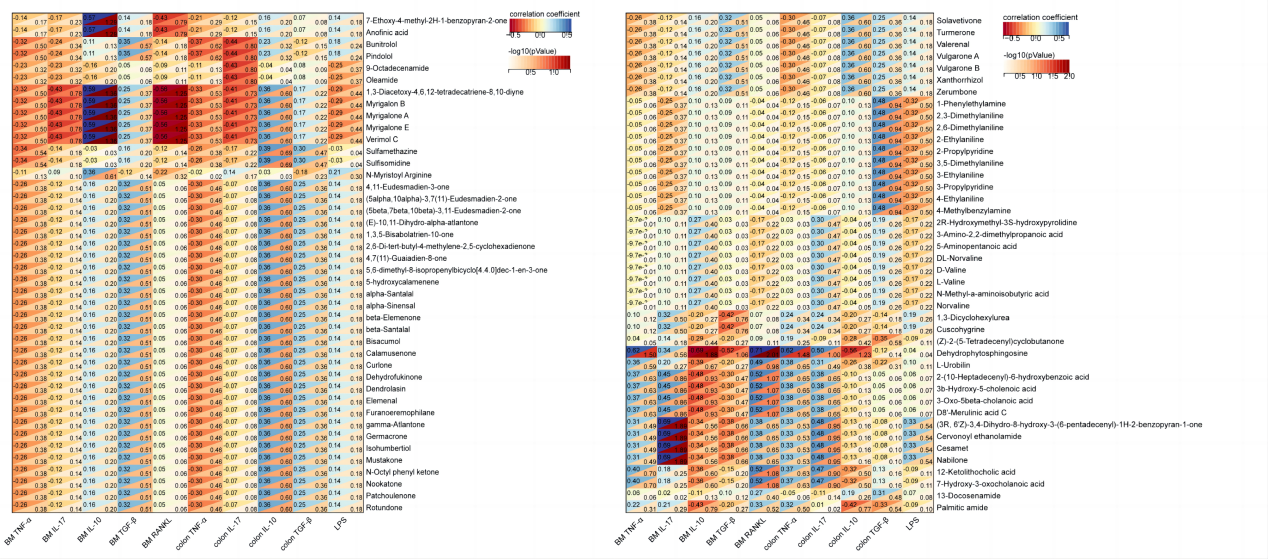


**Supplementary Figure 8.** Correlation of fecal differential metabolites with inflammatory factors. The upper left of each block represents the correlation coefficient, blue represents positive correlation, red represents negative correlation, and the lower right corner represents the correlation, the darker the color, the stronger the correlation.


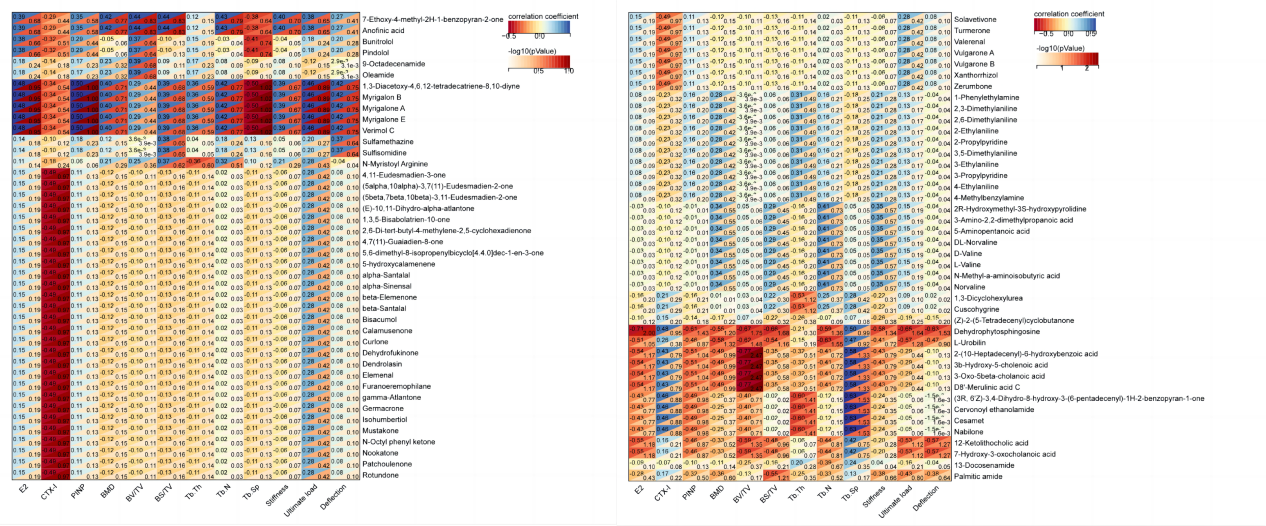


**Supplementary Figure 9.** Correlation of fecal differential metabolites with bone factors. The upper left of each block represents the correlation coefficient, blue represents positive correlation, red represents negative correlation, and the lower right corner represents the correlation, the darker the color, the stronger the correlation.


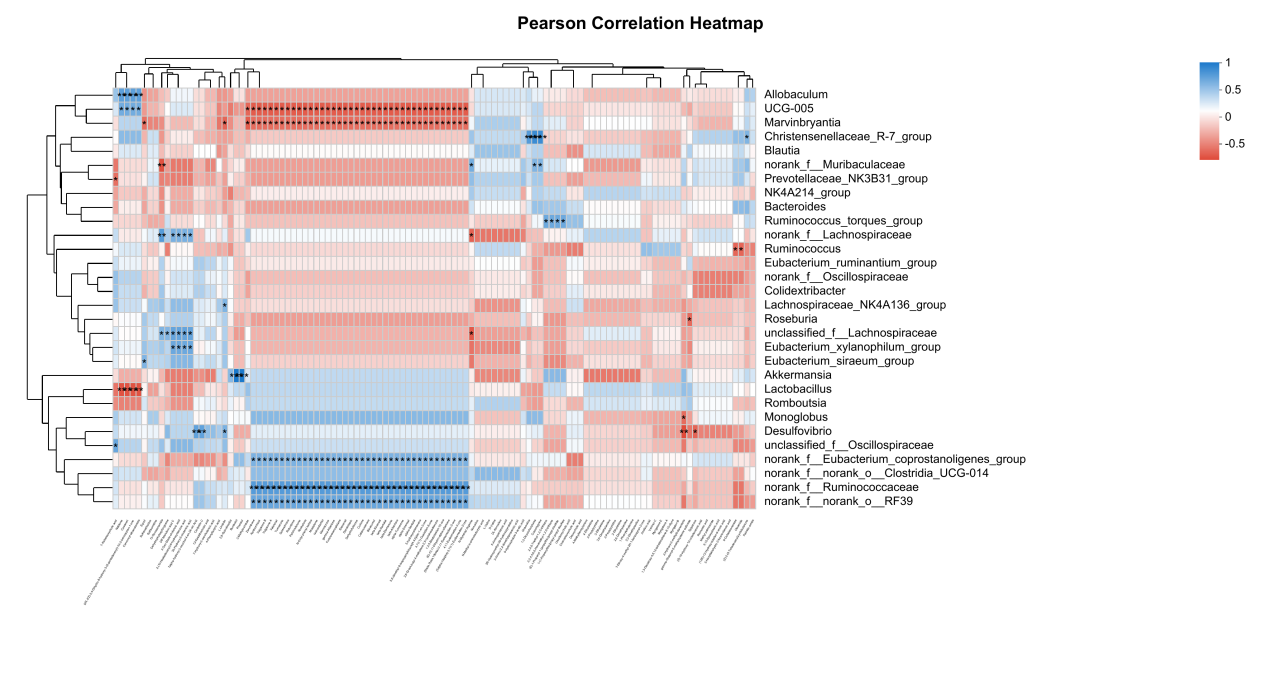


**Supplementary Figure 10.** Correlation of fecal differential metabolites with abundance of the top 30 genera.

Supplementary Table 1 Survival rate of LGG in artificial gastric and intestinal solution.

| Time/h | CFU |
| --- | --- |
| 0 | 3.4 × 10^9^ |
| 2 | 1.0 × 10^9^ |
| 5 | 1.6 × 10^9^ |
| 7 | 1.5 × 10^9^ |
| Survival rate in gastric solution | 29% |
| Survival rate in intestinal solution | 163% |
| Survival rate in artificial digestion solution | 94% |

Supplementary Table 2 Survival rate of LGG in artificial gastric and intestinal solution.

|  | Crypt damage | Goblet cells loss | Inflammatory cell infiltration |
| --- | --- | --- | --- |
| 0 | none | none | none |
| 1 | 0-10% | mild | mild |
| 2 | 10-20% | moderate | moderate |
| 3 | 10-20% | severe | severe |
